# Rab2 regulates presynaptic precursor vesicle biogenesis at the trans-Golgi

**DOI:** 10.1083/jcb.202006040

**Published:** 2021-04-02

**Authors:** Torsten W.B. Götz, Dmytro Puchkov, Veronika Lysiuk, Janine Lützkendorf, Alexander G. Nikonenko, Christine Quentin, Martin Lehmann, Stephan J. Sigrist, Astrid G. Petzoldt

**Affiliations:** 1 Freie Universität Berlin, Institute for Biology and Genetics, Berlin, Germany; 2 Leibniz-Forschungsinstitut für Molekulare Pharmakologie im Forschungsverbund Berlin e.V., Campus Berlin-Buch, Berlin, Germany; 3 Department of Cytology, Bogomoletz Institute of Physiology, Kiev, Ukraine; 4 NeuroCure, Charité, Berlin, Germany

## Abstract

Reliable delivery of presynaptic material, including active zone and synaptic vesicle proteins from neuronal somata to synaptic terminals, is prerequisite for successful synaptogenesis and neurotransmission. However, molecular mechanisms controlling the somatic assembly of presynaptic precursors remain insufficiently understood. We show here that in mutants of the small GTPase Rab2, both active zone and synaptic vesicle proteins accumulated in the neuronal cell body at the trans-Golgi and were, consequently, depleted at synaptic terminals, provoking neurotransmission deficits. Ectopic presynaptic material accumulations consisted of heterogeneous vesicles and short tubules of 40 × 60 nm, segregating in subfractions either positive for active zone or synaptic vesicle proteins and LAMP1, a lysosomal membrane protein. Genetically, Rab2 acts upstream of Arl8, a lysosomal adaptor controlling axonal export of precursors. Collectively, we identified a Golgi-associated assembly sequence of presynaptic precursor biogenesis dependent on a Rab2-regulated protein export and sorting step at the trans-Golgi.

## Teaser

Presynaptic precursors deliver synaptic proteins to nascent and growing synapses. The authors provide evidence that during an early precursor formation step, the small GTPase Rab2 regulates immature precursors formation at the trans-Golgi prior to subsequent Arl8-dependent maturation steps.

## Graphical Abstract

**Figure:**
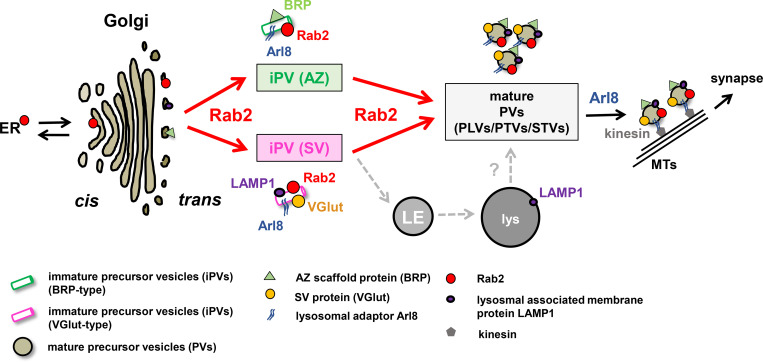

## Introduction

Synaptogenesis is based on the tightly controlled delivery of presynaptic material. Synaptic proteins traffic on presynaptic precursor vesicles from the neuronal soma, where they are produced, to the site of consumption, the synaptic terminal (Ahmari et al., 2000; Chia et al., 2013; Petzoldt et al., 2016; Südhof, 2018). Impairments of presynaptic biogenesis cause severe neurodegenerative or neurodevelopmental diseases, such as Parkinson’s disease, autism spectrum disorder, Alzheimer’s disease, and micro- and macrocephaly (Parrini et al., 2016; Salinas et al., 2008; Waites and Garner, 2011). Different presynaptic cargo proteins are transported along axonal microtubules to the assembling presynapses (Maeder et al., 2014b). This includes active zone (AZ) scaffold proteins, synaptic vesicle (SV) proteins, release factors, and voltage-gated ion channels (Fejtova and Gundelfinger, 2006; Petzoldt et al., 2016; Südhof, 2012). These proteins are thought to traffic in defined stoichiometric ratios as preassembled clusters associated with the precursor membrane to permit effective integration into the nascent presynapse (Ahmari et al., 2000; Goldstein et al., 2008; Siebert et al., 2015; Tao-Cheng, 2007). Despite the fundamental importance of presynapse assembly, the molecular mechanisms underlying presynaptic precursor biogenesis are incompletely understood. The organellar origin and cargo composition of precursors are particularly debated (Chia et al., 2013; Jin and Garner, 2008; Maeder et al., 2014a). Earlier studies suggested that precursors may originate at the Golgi complex (Dresbach et al., 2006; Maas et al., 2012; Shapira et al., 2003). We recently provided evidence that presynaptic precursors possess a lysosomal membrane identity as they acquire lysosomal membrane proteins (LMPs) in a hitherto unexplained maturation process (Vukoja et al., 2018). Transport of these presynaptic lysosome-related vesicles (PLVs) depends on the small Arf-like GTPase Arl8 (Vukoja et al., 2018), a conserved adaptor of kinesin motor proteins implicated in the anterograde transport of lysosomes, lysosome-related organelles (Khatter et al., 2015; Rosa-Ferreira and Munro, 2011), and axonal SV precursors (Klassen et al., 2010).

In the present study, we aim to identify novel regulators of precursor biosynthesis. We hypothesized that precursor biogenesis requires membrane remodeling enzymes, such as Rab (Ras-related in brain) proteins, small GTPases controlling vesicle budding, and fusion via effector protein recruitment (Stenmark, 2009; Zhen and Stenmark, 2015). Rab proteins function as molecular switches by changing between a GTP-bound active state to a GDP-bound inactive state (Stenmark, 2009; Zhen and Stenmark, 2015). We here show that presynaptic precursors originate from the trans-Golgi in an early step of the biosynthetic assembly pathway. This process requires the highly conserved, Golgi-related small GTPase Rab2 for precursor assembly and maturation. Rab2 is reported as a Golgi resident and acts bidirectionally in the ER to Golgi trafficking (Liu and Storrie, 2012; Saraste, 2016; Tisdale and Balch, 1996). In conjunction with the previously shown function of Rab2 and Rab2 effectors in the promotion of dense core vesicle (DCV) maturation in neurons (Ailion et al., 2014; Edwards et al., 2009; Hannemann et al., 2012; Sumakovic et al., 2009) and the association of *rab2* mutations with neurodevelopmental defects, e.g., autism spectrum disorders, schizophrenia (Kiral et al., 2018; Takata et al., 2016), and memory and prefrontal morphology defects (Li et al., 2015), in humans, our data identify Rab2 as a crucial factor in early presynaptic precursor biogenesis.

## Results

We performed a motoneuron-specific RNAi knockdown of neuronally expressed Rab proteins (Chan et al., 2011) analyzing AZ and SV protein distribution in motoneuron somata and terminals of *Drosophila* third instar larvae, identifying exclusively Rab2 as a potential regulator of precursor biogenesis.

### Presynaptic proteins accumulate in the cell bodies of ***rab2***^**−/−**^-deficient neurons

**Figure S1.**
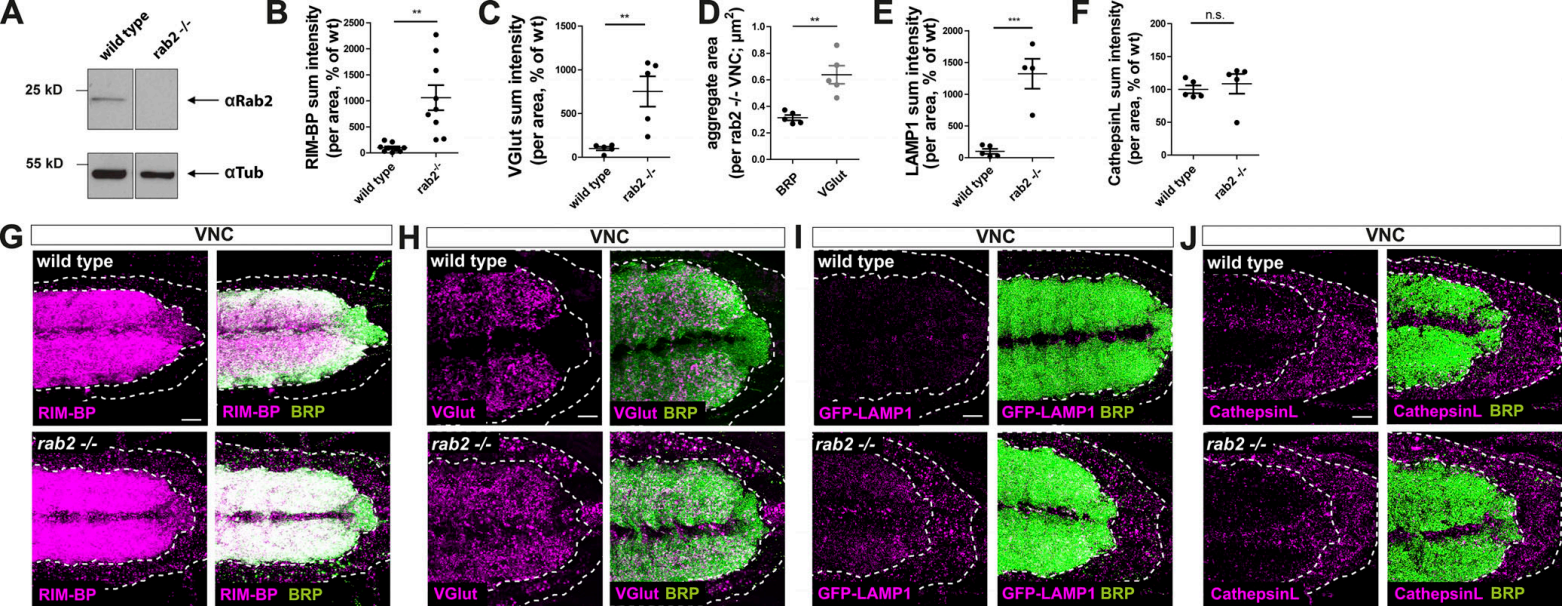
**Presynaptic proteins accumulate in cell bodies of *rab2*^−/−^-deficient neurons.** **(A)** Western blot analysis of wild-type and *rab2*^−/−^ mutant brains probed against Rab2 (top) and α-tubulin (bottom) as loading control. **(B–F)** Quantifications of neuronal somata comparing *rab2*^−/−^ mutant and wild-type. Quantification of RIM-BP sum intensity from Fig. 1 D (B; wild-type: 100.0 ± 26.94%, *n* = 9; *rab2*^−/−^: 1,061 ± 241.8%, *n* = 9) and VGlut sum intensity (C; wild-type: 100.0 ± 21.09%, *n* = 5; *rab2*^−/−^: 752.0 ± 173.4%, *n* = 5) from Fig. 1 F. Quantification of BRP and VGlut aggregate area (D; BRP: 0.314 ± 0.02 nm, *n* = 5; VGlut: 0.638 ± 0.07 nm; *n* = 5) from Fig. 1 F. Quantification of LAMP1 (E; wild-type: 100.0 ± 39.14%, *n* = 5; *rab2*^−/−^: 1,322 ± 235.2%, *n* = 4) from Fig. 1 H and Cathepsin L sum intensity (F; wild-type: 100.0 ± 6.05%, *n* = 5; *rab2*^−/−^: 108.5 ± 14.95%, *n* = 5) from Fig. 1 J. **(G–J)** Confocal images of VNCs deficient for Rab2 (overviews) corresponding to the zooms in Fig. 1, D–J: costained for RIM-BP (G; magenta) and BRP (green), VGlut (H; magenta) and BRP (green), GFP-LAMP1 (I; magenta) and BRP (green), and Cathepsin L (J; magenta) and BRP (green). Scale bar, 10 µm. All graphs show mean ± SEM. *n* represents single VNCs (B–F). Normality was tested with the D’Agostino and Pearson omnibus normality test. If normally distributed (or assumed to be normally distributed for *n* < 7), the unpaired *t* test was used (B–E); otherwise, the nonparametric Mann–Whitney test was used (F). **, P < 0.01; ***, P < 0.001. wt, wild-type.

**Figure 1.**
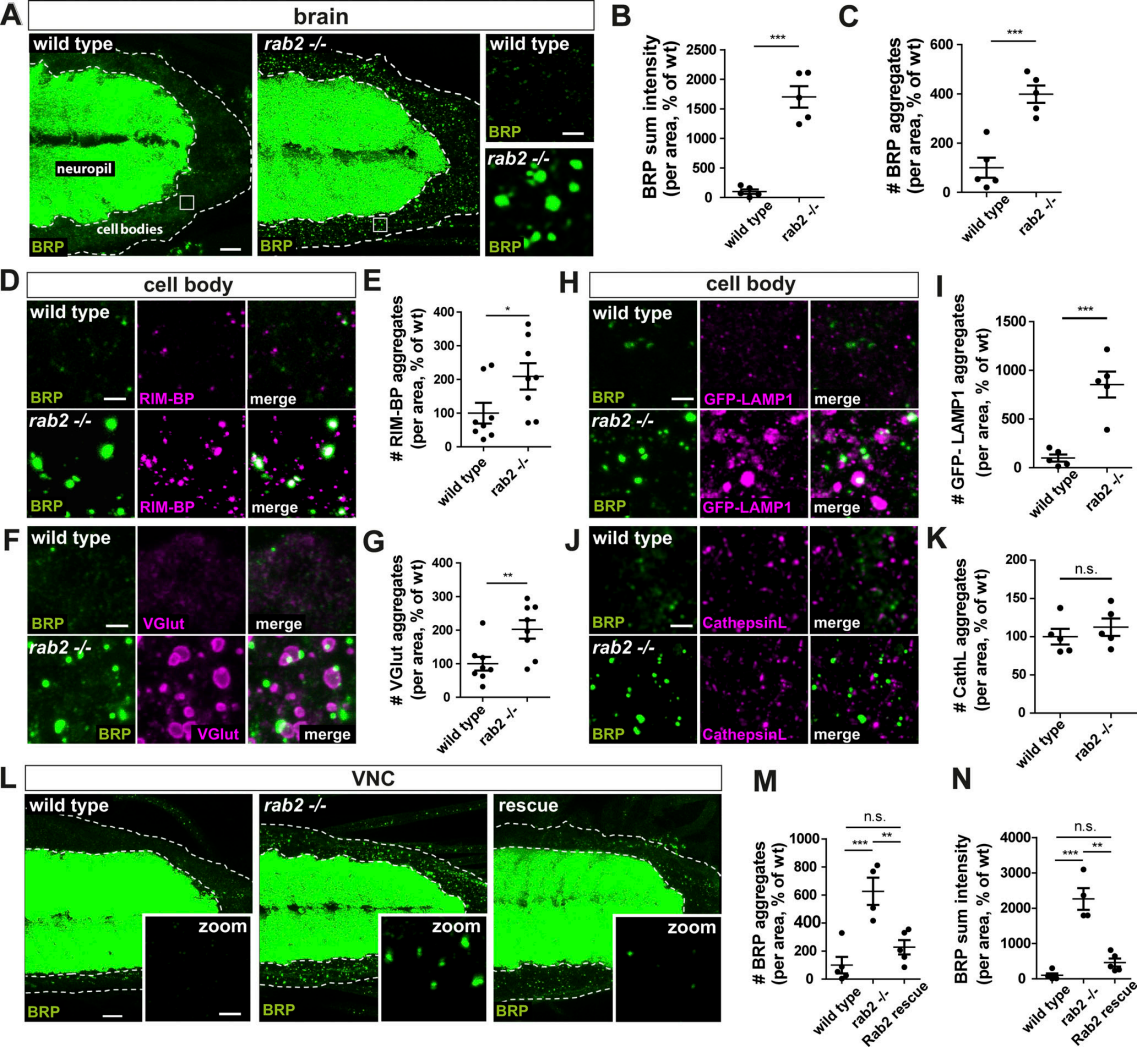
**Presynaptic proteins accumulate in *rab2*^−/−^-deficient neuronal somata.** **(A)** Confocal images of larval VNCs from wild-type and *rab2***^−/−^** mutants labeled for BRP (green). White squares indicate zoom area. **(B)** BRP sum intensity (wild-type: 100.0 ± 36.91%, *n* = 5; *rab2*^−/−^: 1,701 ± 181.7%, *n* = 5). **(C)** Number of BRP aggregates (wild-type: 100.0 ± 40.93%, *n* = 5; *rab2*^−/−^: 398.7 ± 35.27%, *n* = 5). **(D–K)** VNC (zooms) colabeled for BRP (green) and, in magenta, RIM-BP (D), VGlut (F), GFP-LAMP1 (H), and Cathepsin L (J; CathL). Number of RIM-BP aggregates (E; wild-type: 100.0 ± 30.77%, *n* = 8; *rab2*^−/−^: 209.1 ± 39.04%, *n* = 8), VGlut aggregates (G; wild-type: 100.0 ± 20.22%, *n* = 8; *rab2*^−/−^: 202.2 ± 27.43 5%, *n* = 8), LAMP1 aggregates (I; wild-type: 100.0 ± 36.72%, *n* = 5; *rab2*^−/−^: 853.8 ± 133.1%, *n* = 5), and Cathepsin L aggregates (K; wild-type: 100.0 ± 10.32%, *n* = 5; *rab2*^−/−^: 112.4 ± 11.36%, *n* = 5). **(L)** VNC from wild-type, *rab2*^−/−^ mutant, and *rab2*^−/−^ mutants with reexpressed Rab2 labeled for BRP (green). **(M)** Number of BRP aggregates (wild-type: 100.0 ± 58.90%, *n* = 5; *rab2*^−/−^: 626.0 ± 96.90%, *n* = 4; Rab2 rescue: 227.4 ± 51.22%, *n* = 5). **(N)** BRP sum intensity (wild-type: 100.0 ± 53.28%, *n* = 5; *rab2*^−/−^: 2,262.0 ± 308.3%, *n* = 4; Rab2 rescue: 461.0 ± 114.2%, *n* = 5). Scale bars: A and L, 10 µm; zoom (A, D, F, H, J, and L), 2 µm. All data provided as mean ± SEM. *n* represents single VNCs. *, P < 0.05; **, P < 0.01; ***, P < 0.001. wt, wild-type.

**Figure S2.**
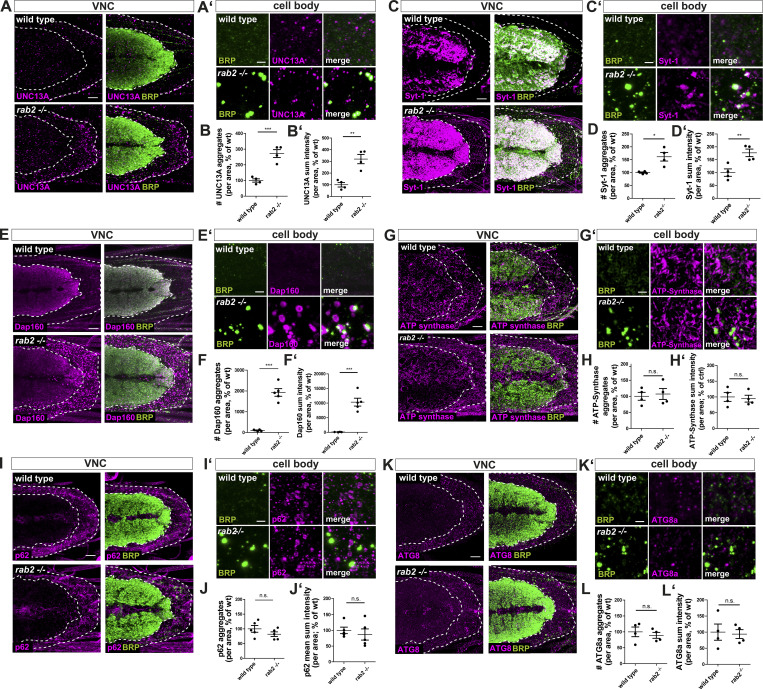
**Presynaptic and nonsynaptic proteins in cell bodies of *rab2*^−/−^-deficient neurons. (A–L)** Confocal images (overviews A–K and zooms A′–K′) of neuronal somata deficient for Rab2 stained for BRP (green) and UNC13A (A, A′; magenta), Syt-1 (C, C′; magenta), Dap160 (E, E′; magenta), ATP-synthase (G, G′; magenta), p62 (I, I′; magenta), and ATG8a (K, K′; magenta). Scale bars, 10 µm; zoom, 2 µm. Quantification of the corresponding number of aggregates for UNC13A (B; wild-type: 100.0 ± 10.66%, *n* = 4; *rab2*^−/−^: 271.6 ± 25.44%, *n* = 4), Syt-1 (D; wild-type: 100.0 ± 2.52%, *n* = 4; *rab2*^−/−^: 161.4 ± 16.69%, *n* = 4), Dap160 (F; wild-type: 100.0 ± 21.24%, *n* = 4; *rab2*^−/−^: 1,927 ± 183.1%, *n* = 5), ATP-synthase (H; wild-type: 100.0 ± 12.38%, *n* = 4; *rab2*^−/−^: 107.2 ± 16.89%, *n* = 4), p62 (J; wild-type: 100.0 ± 11.43%, *n* = 5; *rab2*^−/−^: 81.92 ± 7.939%, *n* = 5), and ATG8a (L; wild-type: 100.0 ± 15.09%, *n* = 4; *rab2*^−/−^: 89.01 ± 9.307%, *n* = 4). Quantification of the corresponding protein sum intensity for UNC13A (B′; wild-type: 100.0 ± 19.18%, *n* = 4; *rab2*^−/−^: 319.7 ± 39.60%, *n* = 4), Syt-1 (D′; wild-type: 100.0 ± 14.46%, *n* = 4; *rab2*^−/−^: 176.5 ± 14.63%, *n* = 4), Dap160 (F′; wild-type: 100 ± 22.5%, *n* = 4; *rab2*^−/−^: 10,300 ± 1,360%, *n* = 5), ATP-synthase (H′; wild-type: 100.0 ± 14.14%, *n* = 4; *rab2*^−/−^: 94.48 ± 10.85%, *n* = 4), p62 (J′; wild-type: 100.0 ± 10.37%, *n* = 5; *rab2*^−/−^: 86.99 ± 17.72%, *n* = 5), and ATG8a (L′; wild-type: 100.0 ± 25.09%, *n* = 4; *rab2*^−/−^: 93.91 ± 13.78%, *n* = 4). All graphs show mean ± SEM. *n* represents single VNCs (B, D, F, H, J, and L). Normality was tested with the D’Agostino and Pearson omnibus normality test. If normally distributed (or assumed to be normally distributed for *n* < 7), the unpaired *t* test was used (B, D, F, H, J, and L). *, P < 0.05; **, P < 0.01; ***, P < 0.001. wt, wild-type.

**Figure S3.**
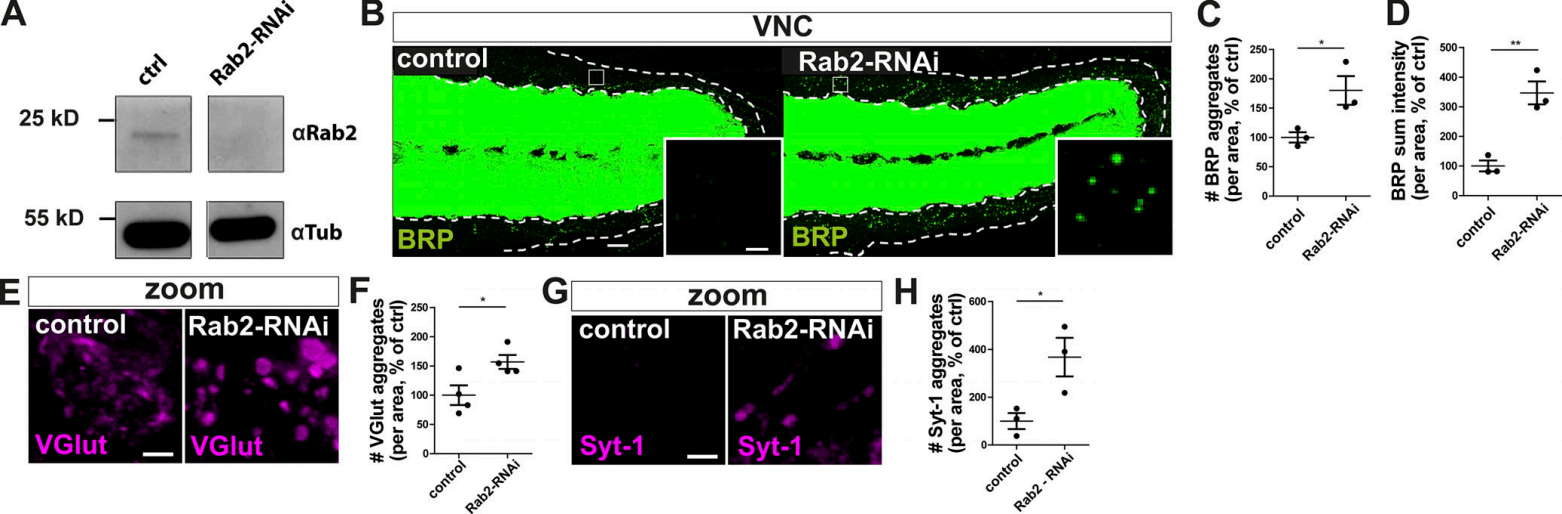
**Presynaptic Rab2 depletion mediated by RNAi knockdown caused accumulation of presynaptic proteins in neuronal cell bodies.** **(A)** Western blot analysis of wild-type and Rab2-RNAi knockdown brains expressing the RNAi specifically in motoneurons (ok6-Gal4 driver) probed against Rab2 (top) and α-tubulin (bottom) as loading control. **(B)** Confocal images of neuronal somata from control (driver control) and Rab2-RNAi knockdown brains immunostained for the AZ scaffold protein BRP (green). Neuronal cell bodies in the VNC cortex (dotted lines). White squares show zoom area. Scale bar, overview, 10 µm; zoom, 2 µm. **(C and D)** Quantifications of the representative images of A. **(C)** Number of BRP aggregates (control: 100.0 ± 8.785%, *n* = 3; Rab2-RNAi: 180.1 ± 24.43%, *n* = 3). **(D)** BRP sum intensity (control: 100.0 ± 18.38%, *n* = 3; Rab2-RNAi: 346.6 ± 38.85%, *n* = 3). **(E)** Confocal images of neuronal somata of control (driver control) and Rab2-RNAi knockdown brains immunostained for BRP (green) and VGlut (magenta). **(F)** Quantifications of number of VGlut aggregates (control: 100.0 ± 16.85%, *n* = 4; Rab2-RNAi: 156.9 ± 11.86%, *n* = 4) of the representative images of E. **(G)** Confocal images of neuronal somata of control (driver control) and Rab2-RNAi knockdown brains immunostained for BRP (green) and Syt-1 (magenta). **(H)** Quantifications of number of Syt-1 aggregates (control: 100.0 ± 33.50%, *n* = 3; Rab2-RNAi: 367.6 ± 80.73%, *n* = 3) of the representative images of G. Scale bar for E and G, 2 µm. All graphs show mean ± SEM. *n* represents single VNCs (C and D) or single NMJs (one or two NMJs/larvae) for F and H. Normality was tested with the D’Agostino and Pearson omnibus normality test. If normally distributed (or assumed to be normally distributed for *n* < 7), the unpaired *t* test was used (C, D, F, H). *n* represents one larval brain from one animal. *, P < 0.05; **, P < 0.01. ctrl, control.

Subsequently, we used a *rab2*^−/−^ mutant created by ORF excision (Chan et al., 2011) to establish a complete loss-of-function background. Mutant Rab2 protein level was evaluated by Western blot analysis (Fig. S1 A), proving the absence of detectable Rab2 protein in the *rab2*^−/−^ null mutant. Mutant animals died at the early third larval stage, consistent with *rab2*^−/−^ null mutants previously described (Lőrincz et al., 2017). We performed immunofluorescence analysis in motoneuron cell bodies located in the cortex of larval ventral nerve cords (VNC) of *rab2*^−/−^ mutants. We detected a striking ectopic accumulation of the AZ scaffold protein Bruchpilot (BRP; Kittel et al., 2006; Wagh et al., 2006) with a strong increase of BRP protein level (Fig. 1, A and B). BRP did not accumulate diffusely in the cytoplasm, but in distinct, large aggregates not observed in controls (Fig. 1 A, zoom; and Fig. 1 C). We further analyzed the *rab2*^−/−^ mutants for a set of presynaptic proteins, including another AZ scaffold protein, RIM-binding protein (RIM-BP; Liu et al., 2011). RIM-BP also accumulated in *rab2*^−/−^ mutant somata (Fig. 1, D and E; and Fig. S1, B and G), and the large, ectopic RIM-BP accumulations overlapped with the BRP signal, while smaller RIM-BP dots also observed in the control were not BRP-positive. The same phenotype was observed for the release factor (m)UNC13A (Böhme et al., 2016; Fig. S2, A and B). Furthermore, SV proteins, here the vesicular glutamate transporter protein (VGlut; Santos et al., 2009), were also strongly accumulating in the *rab2*^−/−^ motoneuron somata (Fig. 1, F and G; and Fig. S1, C and H). However, in contrast to RIM-BP or UNC13A, VGlut aggregates were 2.5-fold larger than BRP aggregates (Fig. S1 D) and, interestingly, localized rather adjacent to the BRP accumulations. Other SV proteins, Synaptotagmin-1 (Syt-1; Zhou et al., 2017; Fig. S2, C and D) and Dap160/intersectin (Pechstein et al., 2010; Fig. S2, E and F), accumulated with a similar distribution. RNAi mediated knockdown of Rab2 verified by Western blot analysis (Fig. S3 A) consistently provoked significant ectopic accumulations of both AZ and SV proteins (BRP, VGlut, and Syt-1) in motoneuronal cell bodies (Fig. S3, B–H). Unlike SV and AZ proteins, levels of the endogenous mitochondrial ATP synthase, which also traffics from the cell body to the neuronal terminal, were not increased in *rab2*^−/−^ mutant motoneuronal somata (Fig. S2, G and H), implying that loss of Rab2 could specifically impair presynaptic protein export, while other trafficking pathways are not affected.

We showed previously that axonally trafficking BRP-positive precursors are copositive for the lysosomal-associated membrane protein LAMP1 (Vukoja et al., 2018). We tested for LAMP1 protein levels in *rab2*^−/−^ mutants by expressing a GFP-LAMP1 construct (Pulipparacharuvil et al., 2005) in motoneurons and observed a strong increase of LAMP1 in *rab2*^−/−^ mutant cell bodies (Fig. 1, H and I; and Fig. S1, E and I). Similar to SV proteins, LAMP1-positive aggregates were larger and localized rather adjacent to ectopic BRP accumulations. Our findings agree with previous observations showing an increase of LAMP1 signal in larval brains of *rab2*^−/−^ mutant (Lund et al., 2018). Importantly, the ectopic presynaptic protein accumulations of *rab2*^−/−^-deficient cells were not positive for standard markers of the lysosomal and autophagosomal system: Cathepsin L (Fig. 1, J and K; and Fig. S1, F and J), p62 (Fig. S2, I and J), or ATG8a (Fig. S2, K and L). Thus, presynaptic protein accumulations of *rab2^−/−^*-deficient neurons are apparently not representing generic degradative compartments.

Finally, we could rescue the BRP accumulation phenotype back to wild-type BRP levels by reexpression of Rab2 at endogenous protein levels into *rab2*^−/−^ mutants (Fig. 1, L–N), proving the cell-autonomous nature of the *rab2*^−/−^ phenotype.

### Presynaptic biogenesis relies on the Rab2-dependent delivery of presynaptic material

**Figure 2.**
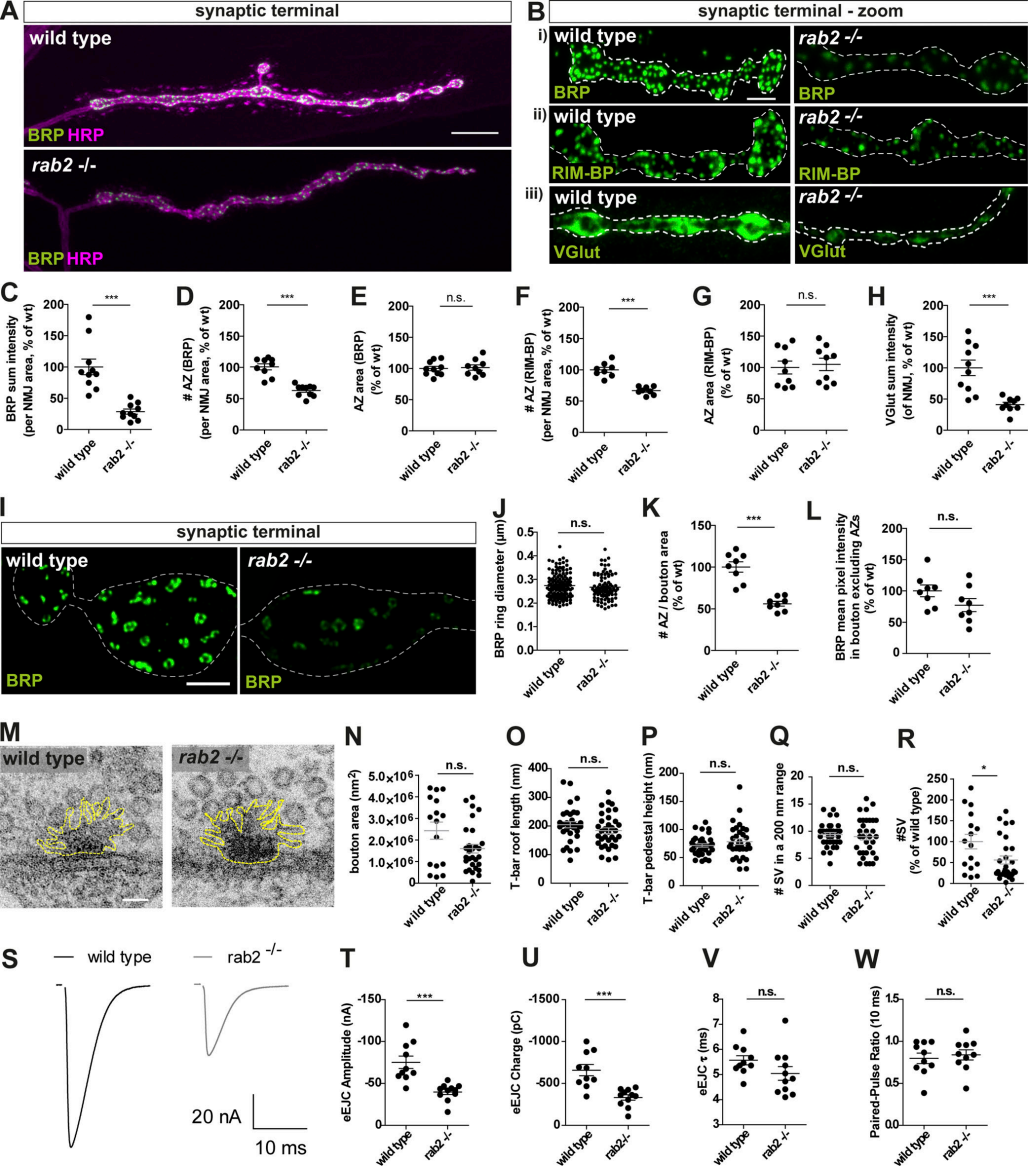
**Presynaptic biogenesis relies on Rab2-dependent delivery of presynaptic material.** **(A and B)** Confocal images of *rab2*^−/−^ mutant NMJs of wild-type and *rab2*^−/−^ mutant animals stained for BRP (A and Bi; green) and HRP (A; magenta), RIM-BP (Bii; green), and VGlut (Biii; green). **(C)** BRP sum intensity (wild-type: 100.0 ± 12.55%, *n* = 10; *rab2*^−/−^: 28.61 ± 4.12%, *n* = 10). **(D)** Number of BRP labeled AZs per NMJ area (wild-type: 100.8 ± 4.87%, *n* = 9; *rab2*^−/−^: 63.06 ± 2.88%, *n* = 10). **(E)** Mean area of individual AZs (wild-type: 100.0 ± 3.55%, *n* = 10; *rab2*^−/−^: 101.6 ± 4.31%, *n* = 9). **(F)** Number of AZs per NMJ area labeled with RIM-BP (wild-type: 100.0 ± 4.05%, *n* = 8; *rab2*^−/−^: 66.69 ± 2.28%, *n* = 8). **(G)** Mean area of individual AZs (wild-type: 100.0 ± 10.29%, *n* = 9; *rab2*^−/−^: 105.0 ± 9.86%, *n* = 9). **(H)** VGlut sum intensity per NMJ area (wild-type: 100.0 ± 12.41%, *n* = 10; *rab2*^−/−^: 40.82 ± 3.92%, *n* = 9). **(I)** STED microscopy images of wild-type and *rab2*^−/−^ mutant NMJs labeled for BRP (green). **(J)** BRP ring diameter (wild-type: 0.273 ± 0.052 µm, *n* = 135; *rab2*^−/−^: 0.267 ± 0.053, *n* = 89). **(K)** Number of AZs per bouton area (wild-type: 100.0 ± 6.29, *n* = 8; *rab2***^−/−^**: 56.07 ± 2.83, *n* = 8). **(L)** Cytoplasmic BRP level outside of AZs (wild-type: 100.0 ± 9.27, *n* = 8; *rab2*^−/−^: 77.36 ± 10.20, *n* = 8). **(M)** Electron micrographs of wild-type and *rab2*^−/−^ mutant AZs. **(N)** Bouton area (wild-type: 2.4 × 10^6^ ± 4 × 10^5^ nm^2^, *n* = 16; *rab2*^−/−^: 1.6 × 10^6^ ± 2 × 10^5^ nm^2^, *n* = 29). **(O)** T-bar roof length (wild-type: 204.2 ± 10.65 nm, *n* = 33; *rab2*^−/−^: 183.7 ± 10.13 nm, *n* = 34). **(P)** T-bar pedestal height (wild-type: 71.58 ± 3.04 nm, *n* = 33; *rab2***^−/−^**: 78.24 ± 5.04 nm, *n* = 34). **(Q)** Number of SVs within 200 nm of T-bar center (wild-type: 9.36 ± 0.39, *n* = 33; *rab2*^−/−^: 9.00 ± 0.61, *n* = 32). **(R)** Number of SVs in percentage of wild-type (wild-type: 100.00 ± 17.41, *n* = 16; *rab2*^−/−^: 55.92 ± 9.5, *n* = 28). **(S–W)** Two electrode voltage-clamp electrophysiological recordings. **(S)** eEJC traces of wild-type and *rab2*^−/−^ mutant synapses. eEJC amplitudes (T; wild-type: −75.14 ± 7.42 nA, *n* = 10; *rab2*^−/−^: −39.82 ± 3.05 nA, *n* = 11), eEJC charge (U; wild-type: −657.7 ± 66.8 pC, *n* = 10; *rab2*^−/−^: −331.8 ± 31.78 pC, *n* = 11), eEJC τ (V; wild-type: 5.57 ± 0.19 ms, *n* = 10; *rab2*^−/−^: 5.04 ± 0.27 ms, *n* = 11), and 10 ms PP ratio (W; wild-type: 0.797 ± 0.063, *n* = 10; *rab2***^−/−^**: 0.839 ± 0.06, *n* = 10). Scale bars: A, 10 µm; B, 3 µm; I, 1 µm; and M, 50 nm. All data provided as mean ± SEM. *n* represents single NMJs (C–H and T–W, one or two NMJs/animal) or single AZs or T-bars (J and O–Q, of three to six animals) or boutons (K, L, N, and R, of three to six animals). *, P < 0.05; ***, P < 0.001. wt, wild-type.

**Figure S4.**
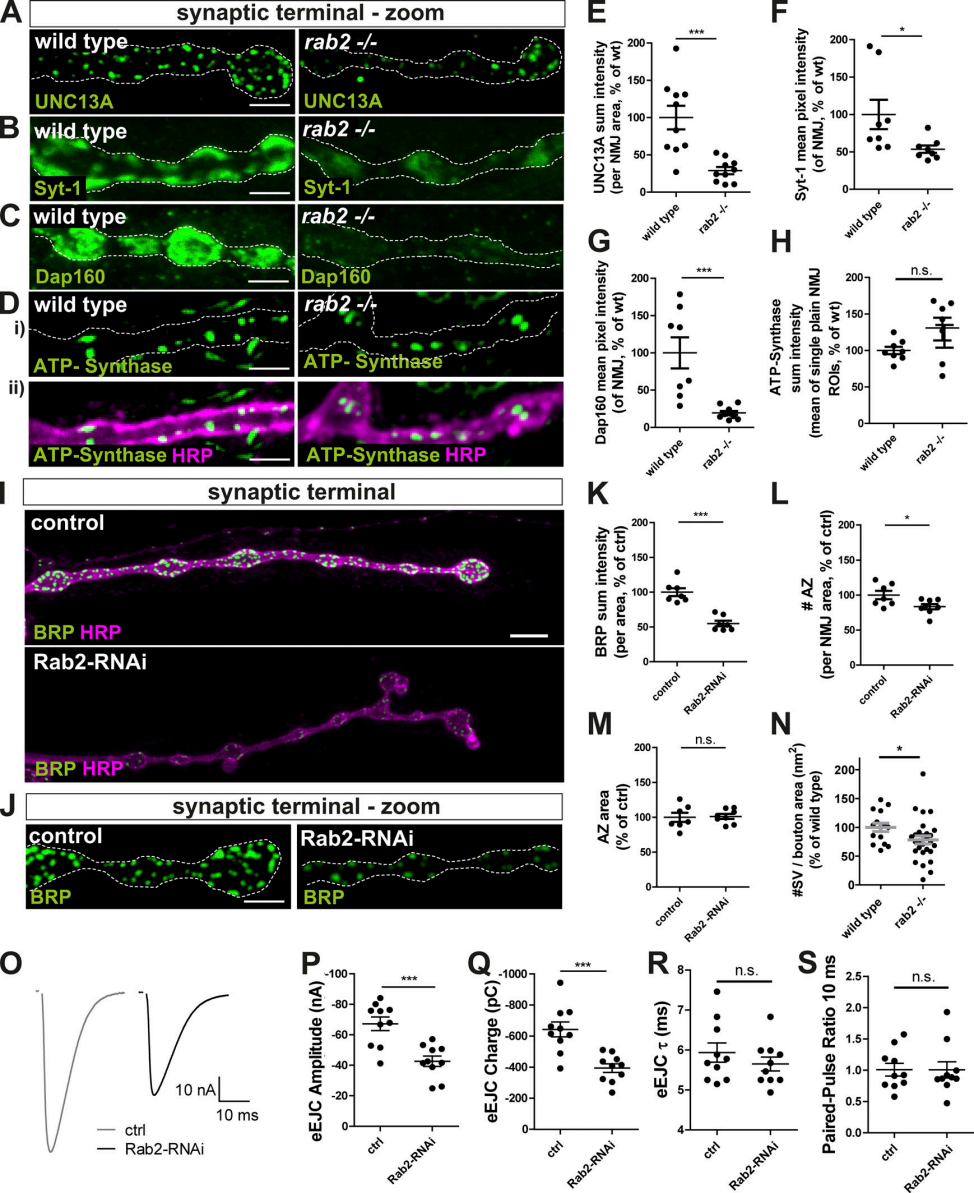
**Presynaptic biogenesis relies on the Rab2 dependent delivery of presynaptic material. (A–H)** Confocal images of wild-type and *rab2*^−/−^ mutant NMJs stained in green for UNC13A (A), Syt-1 (B), Dap160 (C), ATP-synthase (D; green), and HRP (magenta, as a neuronal membrane marker). Scale bar, 3 µm. Corresponding quantification of the protein sum (or mean pixel) intensity for UNC13A (E: wild-type: 100.0 ± 15.83%, *n* = 10; *rab2*^−/−^: 28.96 ± 4.821%, *n* = 10), Syt-1 (F; wild-type: 100.0 ± 19.60%, *n* = 8; *rab2*^−/−^: 53.57 ± 4.89%, *n* = 8), Dap160 (G; wild-type: 100.0 ± 20.83%, *n* = 8; *rab2*^−/−^: 19.35 ± 2.592%, *n* = 10), and ATP-synthase (H; wild-type: 100.0 ± 5.060%, *n* = 8; *rab2*^−/−^: 129.4 ± 15.54%, *n* = 7). **(I)** Confocal images of NMJs from control (driver control) and Rab2-RNAi knockdown terminals immunostained for the AZ scaffold protein BRP (green) and HRP (magenta). **(J)** Corresponding zoom images. Scale bar, overview, 5 µm; zoom, 2 µm. **(K–M)** Quantifications of the representative images of I. **(K)** BRP sum intensity (control: 100.0 ± 5.74%, *n* = 7; Rab2-RNAi: 54.92 ± 4.04%, *n* = 7). **(L)** Number of AZ (control: 100.0 ± 6.0%, *n* = 7; Rab2-RNAi: 83.43 ± 3.772%, *n* = 8). **(M)** AZ area (control: 100.0 ± 6.436%, *n* = 7; Rab2-RNAi: 101.2 ± 3.556%, *n* = 8). **(N)** Number of SVs per bouton area (wild-type: 100.00 ± 7.19, *n* = 15; *rab2*^−/−^: 78.16 ± 7.01, *n* = 28).** (O–S) **Two electrode voltage-clamp electrophysiological recordings of NMJs from control (driver control) and Rab2-RNAi knockdown larvae. **(O)** Representative example eEJC traces of control and RAB2 knockdown synapses. **(P)** eEJC amplitudes (control: −67.18 ± 4.48 nA, *n* = 10; Rab2-RNAi: −42.61 ± 3.408 nA %, *n* = 10). **(Q)** eEJC charge (control: −642.7 ± 48.07 pC, *n* = 10; Rab2-RNAi: −393.9 ± 27.95 pC, *n* = 10). **(R)** eEJC τ (control: 5.94 ± 0.24 ms, *n* = 10; Rab2-RNAi: 5.65 ± 0.17 ms, *n* = 10). **(S)** 10 ms PP ratio (control: 1.01 ± 0.11, *n* = 10; Rab2-RNAi: 1.01 ± 0.13, *n* = 10). All graphs show mean ± SEM. *n* represents single NMJs (one or two per animal). Normality was tested with the D’Agostino and Pearson omnibus normality test. If normally distributed, the unpaired *t* test (E, G, H, K, L, M, N, and P–R) was used. Otherwise, the nonparametric Mann–Whitney test (F and S) was used. *, P < 0.05; ***, P < 0.001. ctrl, control; wt, wild-type.

Assuming that the accumulation of presynaptic proteins in the neuronal somata reflected deficits in assembly or transport of precursor cargo proteins, a consequent reduction of presynaptic proteins at motoneuron synaptic terminals is expected. Indeed, when analyzing neuromuscular terminals of *rab2*^−/−^ mutants, we observed that the entire synaptic terminal of *rab2*^−/−^ mutants appeared thinner with atypically small boutons, phenocopying larvae lacking kinesin Unc104/KIF1a (Pack-Chung et al., 2007; Zhang et al., 2016), or Arl8 (Vukoja et al., 2018). Overall BRP protein levels at *rab2*^−/−^ mutant synaptic terminals were strongly reduced compared with wild-type (Fig. 2 A; Fig. 2 Bi; and Fig. 2 C), and AZ numbers were reduced (Fig. 2 D). However, AZ size remained unaltered (Fig. 2 E), indicating that presynapse assembly per se was not affected due to Rab2 absence. Apart from BRP, also other presynaptic proteins were reduced at *rab2*^−/−^ mutant terminals: RIM-BP (Fig. 2 Bii; and Fig. 2, F and G), UNC13A (Fig. S4, A and E), VGlut (Fig. 2 Biii; and Fig. 2 H), Syt-1 (Fig. S4, B and F), and DAP160 (Fig. S4, C and G). Importantly, mitochondrial ATP synthase was again not altered at synaptic terminals (Fig. S4, D and H), indicating that axonal transport is not generically affected by absence of Rab2. Restricted Rab2 knockdown to motoneuron via RNAi caused a reduction of BRP levels (Fig. S4, I–K) and AZ number (Fig. S4 L) similar to *rab2*^−/−^ mutants, while AZ size was again unaffected (Fig. S4 M). We further evaluated AZ architecture by superresolution stimulated emission depletion (STED) microscopy. The typical BRP C-terminal ring indicative of regular BRP incorporation and topology of the AZ scaffold (Fouquet et al., 2009) appeared normal in *rab2*^−/−^ mutants (Fig. 2 I). BRP ring diameter showed a trend for a reduction but was not significantly altered (Fig. 2 J), and AZ number per bouton area was decreased at *rab2*^−/−^ mutant (Fig. 2 K). We did not detect any diffuse BRP within the presynaptic cytoplasm, as would be expected in the case where BRP cargo would fail to incorporate into AZs (Fig. 2 L). Finally, we performed ultrastructural analyses by EM (Fig. 2 M). Bouton cross-sectional areas showed a trend for reduction in *rab2*^−/−^ mutants (Fig. 2 N), and T-bar roof length and pedestal height were not significantly altered (Fig. 2, O and P). Moreover, SV numbers in proximity to the T-bar were not changed (Fig. 2 Q). Notably, overall numbers of SVs were reduced (Fig. 2 R), as well as numbers of SVs per bouton area (Fig. S4 N), consistent with a reduction of presynaptic SV proteins delivered (Fig. 2 Biii; and Fig. 2 H). Taken together, data from all resolution levels collectively indicate that Rab2 is primarily implied in the supply of biosynthetic presynaptic material to the synaptic terminal and not in the regulation of “acute” protein incorporation at assembling presynapses.

Two-electrode voltage clamp (TEVC) electrophysiological recordings showed that single action potential evoked response was reduced to half of the evoked excitatory junctional currents (eEJCs) in *rab2*^−/−^ mutants (Fig. 2, S and T) and, correspondingly, evoked charge transfer (Fig. 2 U), consistent with the AZ numbers being reduced. However, eEJC kinetics (Fig. 2 V) and short-term plasticity (10 ms paired pulse [PP]; Fig. 2 W) were altered. Rab2-RNAi–mediated knockdown yielded similar results to the *rab2*^−/−^ mutant: eEJC amplitude (Fig. S4, O and P) and eEJC charge (Fig. S4 Q) were significantly reduced, while both kinetics (Fig. S4 R) and short-term plasticity (Fig. S4 S) remained unaltered. Thus, reduction of presynaptic material available at the synaptic terminal in the absence of Rab2 clearly restricts neurotransmitter release. However, those AZs still forming remain apparently functional, in accordance with the overall normal AZ architecture confirmed by microscopy.

### Rab2 is a component of anterogradely trafficking presynaptic precursors

**Figure 3.**
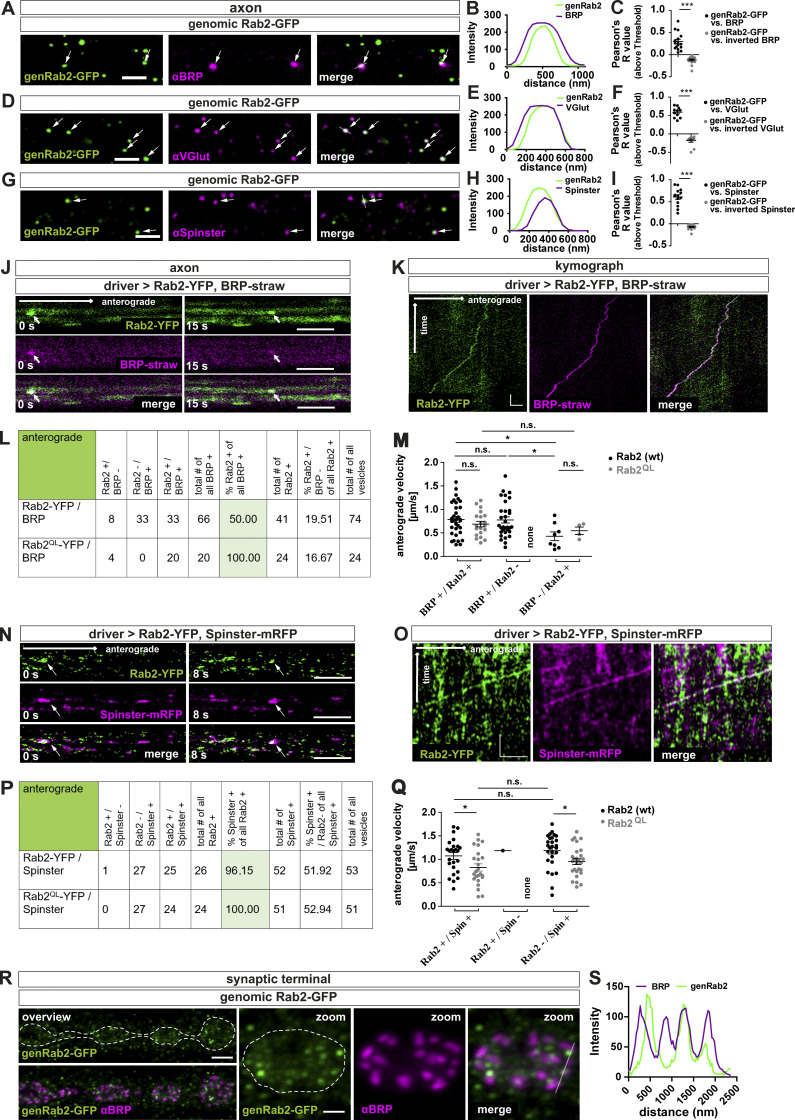
**Rab2 is a component of anterogradely trafficking presynaptic precursors.** **(A–I) **Confocal images of motoneuronal axons in Rab2-expressing larvae (genomically tagged, genRab2-GFP) stained for genRab2-GFP (green) and αBRP (A; magenta), αVGlut (D), and αSpinster (G). Arrows indicate copositive vesicles. **(B, E, and H)** Corresponding line profiles for A, D, and G with the transparent line indicating site of extraction. **(C, F, and I)** Corresponding Pearson’s correlation coefficients: genRab2-GFP versus BRP: 0.3 ± 0.05, *n* = 16; inverted BRP: −0.12 ± 0.02, *n* = 16 (C); genRab2-GFP versus VGlut: 0.59 ± 0.04, *n* = 11; inverted VGlut: −0.18 ± 0.04, *n* = 11 (F); genRab2-GFP versus Spinster: 0.61 ± 0.05, *n* = 14; inverted Spinster: −0.07 ± 0.02, *n* = 14 (I). **(J and K)** In vivo imaging of larvae expressing Rab2-YFP. **(J–Q)** Still images of anterogradely trafficking precursors (arrows) in larvae coexpressing Rab2-YFP (green) and BRP^straw^ (J; magenta) at 0 s or 15 s, and Spinster^mRFP^ (N; magenta) at 0 s and 8 s. Corresponding kymographs, K for J and O for N. Table with quantification of anterograde trafficking presynaptic transport vesicles positive for Rab2-YFP/Rab2^QL^-YFP and BRP^straw^ (L) or Spinster^mRFP^ (P) and corresponding vesicles velocities (M) for BRP^straw^ (BRP^+^/Rab2-YFP^+^: 0.79 ± 0.06 µm/s, *n* = 33; BRP^+^/Rab2^QL^-YFP^+^: 0.69 ± 0.06 µm/s, *n* = 20; BRP^+^/Rab2-YFP^-^: 0.78 ± 0.07 µm/s, *n* = 32; BRP^+^/Rab2^QL^-YFP^−^: no events; BRP^-^/Rab2-YFP^+^: 0.43 ± 0.09 µm/s, *n* = 8; BRP^-^/Rab2^QL^-YFP^+^: 0.54 ± 0.08 µm/s, *n* = 4) and (Q) for Spinster^mRFP^ (Rab2-YFP^+^/Spinster^mRFP+^: 1.07 ± 0.07 µm/s, *n* = 25; Rab2^QL^-YFP^+^/Spinster^mRFP+^: 0.83 ± 0.08 µm/s, *n* = 24; Rab2-YFP^+^/Spinster^mRFP-^: 1.185 µm/s, *n* = 1; Rab2^QL^-YFP^+^/Spinster^mRFP−^: no events; Rab2-YFP^−^/Spinster^mRFP+^: 1.18 ± 0.07 µm/s, *n* = 27; Rab2^QL^-YFP^−^/Spinster^mRFP+^: 0.95 ± 0.06 µm/s, *n* = 27). **(R and S)** Confocal images of NMJs expressing genomic Rab2 stained for genRab2-GFP (green) and BRP (magenta). Dashed line indicates NMJ area, with transparent line indicating site of line profile extraction (S). Scale bars: A, D, and G, 2 µm; J and N, 3 µm; K, 3 µm/10 s; O, 4 µm/20 s; and R, overview 3 µm, zoom 1 µm. All data provided as mean ± SEM. *n* represents single axonal (co)localization (C, F, and I) or transport (L, M, P, N, S, and Q) events in three to six larvae. *, P < 0.05; ***, P < 0.001. wt, wild-type.

**Figure S5.**
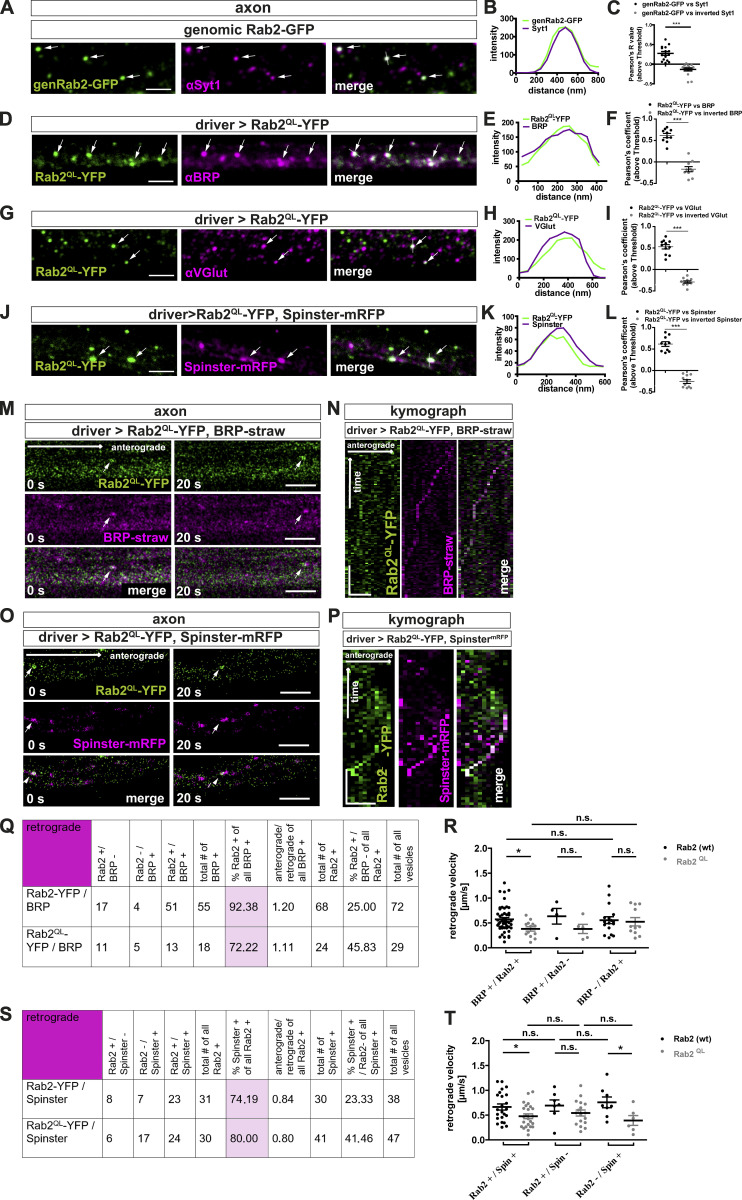
**Rab2 is a component of anterogradely and retrogradely trafficking presynaptic precursors.** **(A–C)** Rab2 colocalized with the SV protein Syt-1 in axons. Confocal images showing presynaptic transport vesicles in axons of third instar *Drosophila* larvae using tagged genomic Rab2 (genRab2-GFP). Immunofluorescent staining showed several puncta (arrows) copositive for genRab2-GFP (green) and Syt-1 (magenta). Scale bar, 2 µm. **(B)** Corresponding line profiles with the transparent line indicating site of extraction. **(C)** Corresponding Pearson’s correlation coefficients: genRab2-GFP versus Syt-1: 0.27 ± 0.05, *n* = 15; inverted Syt-1: −0.13 ± 0.04, *n* = 15. **(D, G, and J)** Overexpression of Rab2^QL^-YFP in third instar *Drosophila* larvae. Immunofluorescent staining showed several puncta (arrows) copositive for Rab2^QL^-YFP (D; green) and αBRP (magenta), for Rab2^QL^-YFP (G; green) and αVGlut, and for Rab2^QL^-YFP (J; green) and overexpressed Spinster^mRFP^. Scale bars, 2 µm. **(E, H, and K)** Corresponding line profiles for D, G, and J, with the transparent line indicating site of extraction. **(F, I, and L)** Corresponding Pearson’s correlation coefficients: (F) Rab2^QL^-YFP versus BRP: 0.62 ± 0.05, *n* = 10; inverted BRP: −0.17 ± 0.06, *n* = 10; (I) Rab2^QL^-YFP versus VGlut: 0.53 ± 0.05, *n* = 11; inverted VGlut: −0.29 ± 0.03, *n* = 11; and (L) Rab2^QL^-YFP versus Spinster^mRFP^: 0.62 ± 0.06, *n* = 10; inverted Spinster^mRFP^: −0.26 ± 0.04, *n* = 10, confirming colocalization. **(M–P)** Anterograde in vivo trafficking of presynaptic transport vesicles copositive for overexpressed Rab2^QL^-YFP and presynaptic proteins or lysosomal marker in vivo. **(M)** Confocal images from live imaged presynaptic transport vesicles trafficking events (arrows) in larvae coexpressing Rab2^QL^-YFP (green) and BRP^straw^ (magenta). Stills at 0 s or 20 s, respectively. Scale bar, 3 µm. **(N)** Kymograph of anterogradely trafficking presynaptic transport vesicles positive for Rab2^QL^-YFP (green) and BRP^straw^ (magenta). Scale bars, 500 nm/20 s. **(O) **Confocal images from live imaged presynaptic transport vesicles trafficking events (arrows) in larvae coexpressing Rab2^QL^-YFP (green) and Spinster^mRFP^ (magenta). Stills at 0 s and 20 s, respectively. Scale bar, 3 µm. **(P)** Kymograph of anterogradely trafficking presynaptic transport vesicles positive for Rab2^QL^-YFP (green) and Spinster^mRFP^ (magenta). Scale bars, 500 nm/20 s. **(Q)** Table with quantification of retrograde transport vesicle trafficking events positive for Rab2-YFP/Rab2^QL^-YFP and BRP^straw^. **(R)** Quantification of transport vesicle velocities (BRP^+^/Rab2-YFP^+^: 0.57 ± 0.04 µm/s, *n* = 50; BRP^+^/Rab2^QL^-YFP^+^: 0.38 ± 0.05 µm/s, *n* = 13; BRP^+^/Rab2-YFP^−^: 0.63 ± 0.16 µm/s, *n* = 4; BRP^+^/Rab2^QL^-YFP^−^: 0.38 ± 0.09 µm/s, *n* = 5; BRP^−^/Rab2-YFP^+^: 0.56 ± 0.07 µm/s, *n* = 17; BRP^−^/Rab2^QL^-YFP^+^: 0.52 ± 0.08 µm/s, *n* = 11). **(S)** Table with quantification of retrograde transport vesicle trafficking events positive for Rab2-YFP/Rab2^QL^-YFP and Spinster^mRFP^. **(T)** Quantification of transport vesicle velocities (Rab2-YFP^+^/Spinster^mRFP+^: 0.66 ± 0.06 µm/s, *n* = 23; Rab2^QL^-YFP^+^/Spinster^mRFP+^: 0.48 ± 0.05 µm/s, *n* = 24; Rab2-YFP^+^/Spinster^mRFP-^: 0.76 ± 0.11 µm/s, *n* = 8; Rab2^QL^-YFP^+^/Spinster^mRFP−^: 0.39 ± 0.1 µm/s, *n* = 6; Rab2-YFP^-^/Spinster^mRFP+^: 0.69 ± 0.11 µm/s, *n* = 7; Rab2^QL^-YFP^−^/Spinster^mRFP+^: 0.54 ± 0.06 µm/s, *n* = 17). All data provided as mean ± SEM. *n* represents the number of axonal (co)localization (C, F, I, and L) or transport (R and T) events in 3–6 larvae. Normality was tested with the D’Agostino and Pearson omnibus normality test. If normally distributed (or assumed to be normally distributed for *n* < 7), the unpaired two-tailed *t* test was used (C, F, I, and L). For R and T, data distribution was tested following the D’Agostino and Pearson omnibus normality test. All data had a normal distribution (or were assumed to be normally distributed for *n* < 7), except for BRP^+^/Rab2^QL^-YFP^+^. We performed for R an unpaired, two-sided Mann–Whitney *U* test comparing the indicated datasets, and for T an unpaired, two-sided *t* test comparing the indicated datasets. *, P < 0.05; ***, P < 0.001. wt, wild-type.

Could Rab2, as a membrane anchored GTPase, localize to in vivo trafficking precursors? By use of a genomic Rab2-GFP construct (genRab2-GFP), with the GFP inserted in the Rab2 ORF (Lund et al., 2018), we colabeled larval axons for BRP and observed distinct Rab2-positive puncta, partially copositive for BRP (Fig. 3 A), also shown by line profile (Fig. 3 B) and Pearson’s correlation coefficient (Fig. 3 C). Similarly, we detected puncta copositive for genRab2-GFP and VGlut (Fig. 3, D–F), Syt-1 (Fig. S5, A–C), or the lysosomal marker Spinster (Rong et al., 2011; Sweeney and Davis, 2002; Fig. 3, G–I). Rab2 in a GTP locked constitutively active form (Rab2^QL^-YFP) expressed in motoneurons showed a similar colocalization profile (Fig. S5, D–L). Next, we directly monitored axonal trafficking in vivo in intact larvae (Andlauer and Sigrist, 2012a), therefore coexpressing strawberry-tagged “BRP short” (BRP^D3^-straw; Fouquet et al., 2009) and Rab2-YFP or constitutively active Rab2^QL^-YFP (Fig. 3, J and K; and Fig. S5, M and N). 50% of anterograde trafficking BRP-positive vesicles were copositive for Rab2 (wild-type) and 100% copositive for constitutively active Rab2^QL^ (Fig. 3, J–L). Thus, Rab2 might be activated during transport. Velocities of Rab2/Rab^QL^ and BRP-positive vesicles were equal (∼0.79 µm/s for Rab2 and ∼0.69 µm/s for Rab2^QL^ or only BRP-positive vesicles [∼0.78 µm/s; Fig. 3 M]). By contrast, trafficking vesicles devoid of BRP moved more slowly (∼0.55 µm/s Rab2/∼0.43 µm/s Rab2^QL^). Retrograde trafficking vesicles were in the majority copositive for BRP and either Rab2 construct (93% for Rab2 [wild-type], 72% for Rab2^QL^; Fig. S5 Q) and slower (∼0.57 µm/s wild-type Rab2/∼0.38 µm/s Rab2^QL^; Fig. S5 R). Also, coexpression of Spinster-mRFP and Rab2-YFP/Rab2^QL^-YFP showed that almost all Rab2-positive vesicles were copositive for Spinster, independent of the Rab2 activity status (96% wild-type/100% Rab2^QL^; Fig. 3, N–P) and moved with a velocity of ∼1.1 µm/s for Rab2 and ∼0.83 µm/s for constitutively active Rab2^QL^ (Fig. 3 Q). Thus, all anterograde trafficking BRP- and Rab2-copositive vesicles are also Spinster-positive, confirming that Rab2, probably in its activated form, is a component of mature presynaptic precursors traveling to the synaptic terminal. Retrograde trafficking Rab2-positive vesicles were also mostly copositive for Spinster (74% for wild-type Rab2 and 80% for Rab2^QL^; Fig. S5 S) and slower (∼0.66 µm/s for wild-type Rab2 and ∼0.48 µm/s for Rab2^QL^; Fig. S5 T). As a membrane component of anterogradely trafficking precursor vesicles, we should expect Rab2 to reach the synaptic terminal. Indeed, we observed genRab2-GFP in a punctate pattern at the synaptic terminals, localizing mostly adjacent to the AZ (Fig. 3, R and S). Interestingly, Rab2 might here identify cargo-loaded precursors “waiting to be discharged” and/or vesicles destined for retrograde transport.

In summary, we provided evidence that Rab2 is associated to presynaptic precursors, supporting the hypothesis that ectopic presynaptic protein accumulations in *rab2*^−/−^ mutants consist of immature, trafficking-incompetent precursors.

### Presynaptic precursors derive from the trans-Golgi

**Figure 4.**
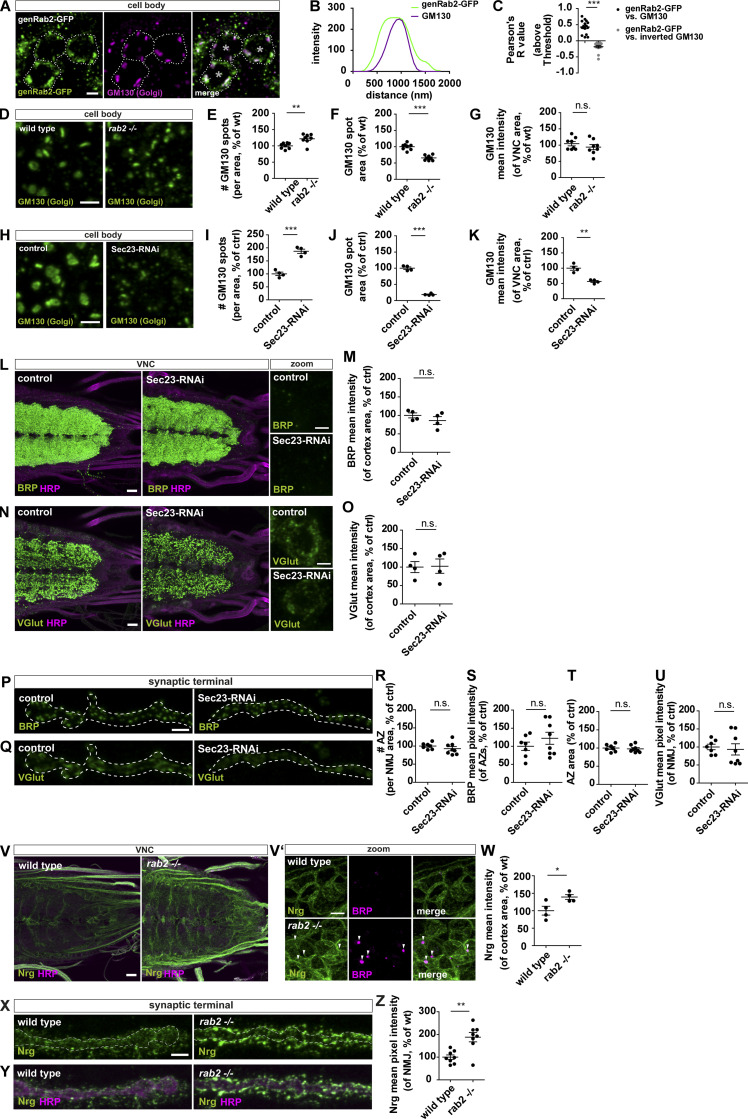
**Disruption of the Golgi is not generically affecting precursor biogenesis.** **(A)** Confocal images of neuronal cell bodies (outlined by dashed line; *, nucleus) expressing genomic Rab2-GFP stained for genRab2 (green) and GM130 (magenta). **(B)** Corresponding line profiles with the transparent line indicating site of extraction. **(C)** Pearson’s correlation coefficient (genRab2-GFP versus GM130: 0.43 ± 0.05, *n* = 15; inverted GM130: −0.19 ± 0.04, *n* = 15). **(D–Q)** Confocal images of neuronal somata of wild-type and *rab2*^−/−^ mutant (D) and control and Sec23-RNAi–expressing larvae labeled for GM130 (H; green). Number of GM130-labeled spots per VNC area for *rab2*^−/−^ mutant (E; wild-type: 100 ± 3.10%, *n* = 8; *rab2*^−/−^: 121.9 ± 5.37%, *n* = 8), and Sec23-RNAi larvae (I; control: 100 ± 6.57%, *n* = 4; Sec23-RNAi: 186.9 ± 8.07%, *n* = 4). GM130-labeled spot area (F; wild-type: 100 ± 3.52%, *n* = 8; *rab2*^−/−^: 65.77 ± 2.84%, *n* = 8), and Sec23-RNAi larvae (J; control: 100 ± 3.85%, *n* = 4; Sec23-RNAi: 18.81 ± 1.43%, *n* = 4). Mean pixel intensity of GM130 signal within the analyzed VNC area for *rab2*^−/−^ mutant (G; wild-type: 104.4 ± 6.64%, *n* = 8; *rab2*^−/−^: 93.76 ± 7.93%, *n* = 8), and Sec23-RNAi larvae (K; control: 100 ± 6.61%, *n* = 4; Sec23-RNAi: 56.59 ± 3.17%, *n* = 4). Confocal images of larval VNCs from control and Sec23-RNAi larvae labeled for BRP (L; green) or VGlut (N; green) and HRP (magenta). Mean pixel intensity for BRP (M; control: 100 ± 6.61%, *n* = 4; Sec23-RNAi: 86.04 ± 10.46%, *n* = 4) and VGlut (O; control: 100 ± 14.88%, *n* = 4; Sec23-RNAi: 102.4 ± 19.42%, *n* = 4). Confocal images of control and Sec23-RNAi NMJs stained for BRP (P; green) or VGlut (Q; green). **(R)** Number of AZs per NMJ area (control: 100 ± 3.21%, *n* = 7; Sec23-RNAi: 92.71 ± 5.85%, *n* = 8). **(S)** BRP mean pixel intensity of individual AZs (control: 100 ± 11.45%, *n* = 7; Sec23-RNAi: 122.0 ± 16.70%, *n* = 8). **(T)** AZ area (control: 100 ± 3.79%, *n* = 7; Sec23-RNAi: 97.50 ± 3.46%, *n* = 8). **(U)** VGlut mean pixel intensity (control: 100 ± 7.44%, *n* = 7; Sec23-RNAi: 93.17 ± 15.55%, *n* = 8). **(V and V′)** Confocal images of wild-type and *rab2*^−/−^ mutant VNCs stained for Nrg (green) and HRP (magenta) or BRP (V′; magenta). Arrowheads point to ectopic BRP aggregates. **(W)** Nrg mean pixel intensity (control: 100 ± 12.62%, *n* = 4; *rab2*^−/−^: 139.1 ± 6.85%, *n* = 4). **(X and Y) **Confocal images of wild-type and *rab2*^−/−^ mutant NMJs stained for Nrg (green) and HRP (magenta). **(Z)** Nrg mean pixel intensity (control: 100 ± 9.77%, *n* = 4; *rab2*^−/−^: 188.0 ± 20.63%, *n* = 4). Scale bars: VNC zooms (A, D, H, L, N, and V′), 2 µm; VNC overviews (L, N, and V), 10 µm; and NMJs (P, Q, X, and Y), 3 µm. All data provided as mean ± SEM. *n* represents single evaluated coaggregates from three VNCs (C), single VNCs (E–G, I–K, M, O, and W), or single NMJs (R–U, Z) with one or two NMJs/animal. *, P < 0.05; **, P < 0.01; ***, P < 0.001. ctrl, control; genRab2, genomic Rab2; wt, wild-type.

**Figure S6.**
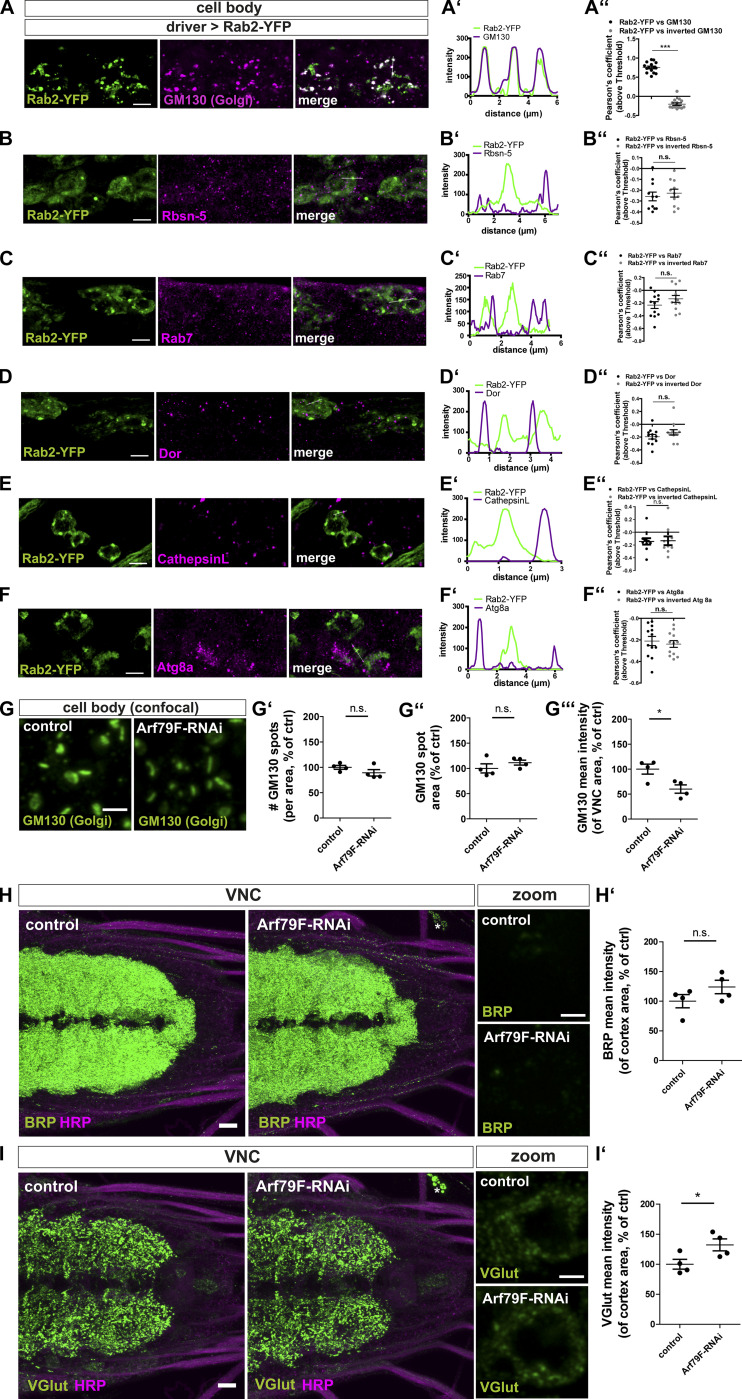
**Rab2 localizes to the Golgi in neuronal somata; disrupted Golgi is not affecting presynaptic precursor biogenesis.** **(A)** Confocal images of neuronal cell bodies (outlined by dashed line) in the brain cortex of third instar larvae expressing Rab2-YFP (green) stained for the Golgi marker GM130 (magenta). Scale bar, 2 µm. **(A′)** Corresponding line profiles with the transparent line indicating site of extraction. **(A″) **Pearson’s correlation coefficient (Rab2-YFP versus GM130: 0.75 ± 0.04, *n* = 15; inverted GM130: −0.21 ± 0.03, *n* = 15) for A. **(B–F)** Confocal images of neuronal somata of larvae expressing Rab2-YFP (green) into the motoneurons costained in magenta for Rbsn-5 (B), Rab7 (C), Dor (D), Cathepsin L (E), and ATG8a (F). Scale bar, 5 µm. Corresponding line profiles with the transparent line indicating site of extraction for Rbsn-5 (B′), Rab7 (C′), Dor (D′), Cathepsin L (E′), and ATG8a (F′). Corresponding Pearson’s correlation coefficients for Rbsn-5 (B″; Rab2-YFP versus Rbsn-5: −0.26 ± 0.04, *n* = 11; inverted Rbsn-5: −0.23 ± 0.04, *n* = 11), Rab7 (C″; Rab2-YFP versus Rab7: −0.23 ± 0.05, *n* = 12; inverted Rab7: −0.13 ± 0.053, *n* = 12), Dor (D″; Rab2-YFP versus Dor: −0.2 ± 0.04, *n* = 12; inverted DOR: −0.13 ± 0.04, *n* = 12), Cathepsin L (E″; Rab2-YFP versus Cathepsin L: −0.14 ± 0.05, *n* = 10; inverted Cathepsin L: −0.13 ± 0.07, *n* = 10), and ATG8a (F″; Rab2-YFP versus ATG8a: −0.21 ± 0.04, *n* = 12; inverted ATG8a: −0.24 ± 0.03, *n* = 12). **(G)** Confocal images of neuronal somata of control and Arf79F-RNAi larvae labeled for the Golgi marker GM130 (green). Scale bar, 2 µm. Quantification of the Golgi properties of representative images in G: number of GM130 labeled spots per VNC area in percentage of wild-type (G′; control: 100 ± 3.75%, *n* = 4; Arf79F-RNAi: 89.21 ± 6.32%, *n* = 4), GM130 labeled spot area in percentage of control (G″; control: 100 ± 8.94%, *n* = 4; Arf79F-RNAi: 111.4 ± 4.88%, *n* = 4), and mean pixel intensity of GM130 signal within the analyzed VNC area in percentage of control (G′′′; control: 100 ± 10.00%, *n* = 4; Arf79F-RNAi: 60.01 ± 8.37%, *n* = 4). **(H and I)** Confocal images of larval VNCs from control and Arf79F-RNAi larvae immunostained for BRP (H; green) and VGlut (I; green) and the neuronal membranes (HRP, magenta) showing no ectopic VGlut aggregates forming. Scale bars, 10 µm; zoom, 2 µm. **(H′)** Quantifications of the representative images of H. BRP mean pixel intensity of the cortex in percentage of control (control: 100 ± 11.12%, *n* = 4; Arf79F-RNAi: 124.0 ± 11.39%, *n* = 4). **(I′)** Quantifications of the representative images of I. VGlut mean pixel intensity of the cortex in percentage of control (control: 100 ± 8.16%, *n* = 4; Arf79F-RNAi: 132.2 ± 9.85%, *n* = 4). All graphs show mean ± SEM. *n* represents single evaluated coaggregates (A″–F″, in three larvae) and single VNCs (G′, G″, G′′′, H′, and I′). Normality was tested with the D’Agostino and Pearson omnibus normality test. If normally distributed (or assumed to be normally distributed for *n* < 7), the unpaired *t* test was used (A″–F″, G′–G′′′, H′, and I′). *, P < 0.05; ***, P < 0.001. ctrl, control.

Using the genRab2-GFP construct, we now thought to identify the site of Rab2 function during precursor biogenesis. Rab2 localized to large organelles in the cytoplasm of neuronal somata, which were frequently copositive for the Golgi marker GM130 (Fig. 4 A), also shown in the line profile (Fig. 4 B) and by a positive Pearson’s correlation coefficient (Fig. 4 C). We observed a similar Rab2 Golgi localization overexpressing Rab2-YFP (Fig. S6 A), while colabeling with markers for the endo-lysosomal system, such as the early endosomal marker Rabenosyn-5 (Rbsn-5), a RAB5-effector protein, (Fig. S6 B) or the late endosomal marker Rab7 (Fig. S6 C), as well as degradative organelles, using the multivesicular body marker deep orange (DOR/VPS18; Fig. S6 D), the lysosomal marker Cathepsin L (Fig. S6 E), or the autophagosomal marker ATG8a (Fig. S6 F), showed not a similarly strong signal overlap. We thus could validate the previously described Golgi localization of Rab2 (Liu and Storrie, 2012; Saraste, 2016; Tisdale and Balch, 1996) in neuronal somata of *Drosophila* motoneurons. However, this does not exclude Rab2 being present in other cellular compartments, as reported previously for other cell types (Ding et al., 2019; Fujita et al., 2017; Lőrincz et al., 2017; Lund et al., 2018).

**Figure S7.**
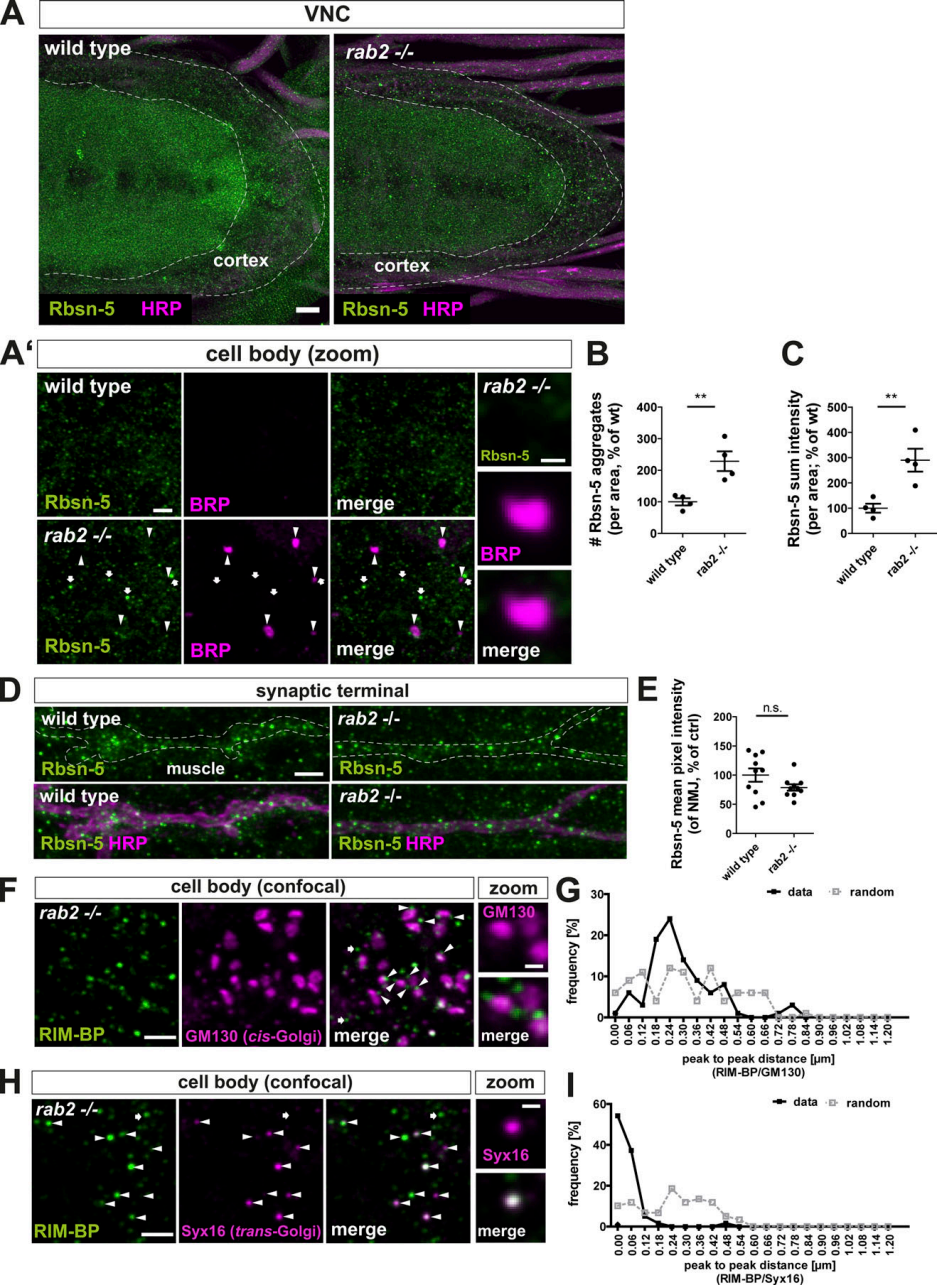
**Golgi sorting and targeting of nonsynaptic proteins is not affected, and RIM-BP accumulates at the trans-Golgi in *rab2*^−/−^ mutants.** **(A and A′) **Confocal images of wild-type and *rab2*^−/−^ mutant neuronal cell bodies in the brain cortex (overview and zooms) of third instar larvae stained for the transsynaptic protein Rbsn-5 (green) and HRP (A; magenta) or BRP (A′; magenta). Arrowheads point to ectopic BRP aggregates, arrows to enlarged Rabsn-5–positive organelles. Scale bars, overview, 10 µm; zoom, 2 µm; mini-zoom, 0.5 µm. Rbsn-5 did not accumulate in ectopic BRP-positive aggregates in the neuronal somata, but Rbsn-5–positive organelles were enlarged and increased in numbers in *rab2*^−/−^ mutants. **(B)** Number of Rbsn-5–positive organelles per area (control: 100 ± 11.30%, *n* = 4; *rab2*^−/−^ mutant: 228.7 ± 31.21%, *n* = 4). **(C)** Rbsn-5 sum intensity per area (control: 100 ± 17.59%, *n* = 4; *rab2*^−/−^ mutant: 290.0 ± 45.39%, *n* = 4). **(D)** Confocal images of wild-type and *rab2*^−/−^ mutant NMJs stained for Rbsn-5 (green) and HRP (magenta). Scale bar, 3 µm. **(E)** Quantification of the representative image in D. Rbsn-5 mean pixel intensity in percentage of wild-type was not altered in *rab2*^−/−^ mutants (control: 100 ± 11.35%, *n* = 4; *rab2*^−/−^ mutant: 78.75 ± 5.5%, *n* = 4). **(F and G)** Presynaptic precursor accumulations of *rab2*^−/−^ mutants localized at the trans-Golgi. Confocal images of neuronal cell bodies costained for RIM-BP (green) and the cis-Golgi marker GM130 (magenta) with arrowheads highlighting adjacent localizations and arrows few nonadjacent events. Zoom shows planar and lateral orientation of the Golgi. Scale bar, overview, 2 µm; zoom, 0.5 µm. **(G)** Frequency of peak-to-peak distance distribution of RIM-BP–positive accumulations and cis-Golgi from F; black line for data and dashed gray line for 80 pixel–shifted images for random distribution (distance, % data [n]/% random [n]: 0.00 nm, 1/6%; 0.06 nm, 6/9%; 0.12 nm, 3/11%; 0.18 nm, 19/4%; 0.24 nm, 24/12%; 0.30 nm, 14/11%; 0.36 nm, 9/4%; 0.42 nm, 6/12%; 0.48 nm, 8/4%; 0.54 nm, 1/6%; 0.60 nm, 0/6%; 0.66 nm, 0/6%; 0.72 nm, 1/0%; 0.78 nm, 3/0%; 0.84 nm, 0/1%; 0.90 nm, 0/0%; 0.96 nm, 0/0%; 1.02 nm, 0/0%; 1.08 nm, 0/0%; 1.14 nm, 0/0%; and 1.20 nm, 0/0%). *n* = 95. **(H)** Confocal images of neuronal cell bodies costained for RIM-BP (green) and the trans-Golgi marker Syx16 (magenta) with arrowheads highlighting overlapping localizations and arrows few nonoverlapping events. Scale bars, overview, 2 µm; zoom, 0.5 µm. **(I)** Frequency of peak-to-peak distance distribution of RIM-BP–positive accumulations and trans-Golgi from H. Black line for data and dashed gray line for 80 pixel–shifted images for random distribution (distance in nm, data [%]/random [%]: 0.00 nm, 54/10%; 0.06 nm, 37/11%; 0.12 nm, 5/6%; 0.18 nm, 1/6%; 0.24 nm, 0/18%; 0.30 nm, 0/11%; 0.36 nm, 0/13%; 0.42 nm, 0/11%; 0.48 nm, 1/5%; 0.54 nm, 0/3%; 0.60 nm, 0/0%; 0.66 nm, 0/0%; 0.72 nm, 0/0%; 0.78 nm, 0/0%; 0.84 nm, 0/0%; 0.90 nm, 0/0%; 0.96 nm, 0/0%; 1.02 nm, 0/0%; 1.08 nm, 0/0%; 1.14 nm, 0/0%; and 1.20 nm, 0/0%). *n* = 98. All graphs show mean ± SEM. *n* represents single VNCs (B and C), single NMJs (one or two NMJs/larvae; E), or single peak-to-peak measurements from three VNCs (G and I). Normality was tested with the D’Agostino and Pearson omnibus normality test. If normally distributed (or assumed to be normally distributed for *n* < 7), the unpaired *t* test was used (B, C, and E). *, P < 0.05; **, P < 0.01.

Rab2 is described as a Golgi resident, and its depletion is reported to affect Golgi apparatus integrity (Aizawa and Fukuda, 2015; Maringer et al., 2016). Indeed, the number of Golgi complexes (measured by GM130) was increased by 20% (Fig. 4 E), while Golgi area was decreased by 35% (Fig. 4 F) in *rab2*^−/−^ mutants, pointing toward a Golgi fragmentation defect. GM130 protein level was not altered (Fig. 4 G). As *rab2*^−/−^ mutants showed an altered Golgi architecture, it is conceivable that generic protein biogenesis or general post-Golgi sorting defects could cause the ectopic presynaptic protein accumulation we observed at the trans-Golgi. To address this question, we first used RNAi lines targeting proteins of the coat protein II and I (COPII and COPI) complexes, implied in Golgi biogenesis, maintenance, and consequently function (Kondylis and Rabouille, 2009; Popoff et al., 2011; Ward et al., 2001), thus generically disrupting Golgi function. We restricted protein knockdown to the motoneurons to circumvent survival deficits. If generic Golgi disruption accounts for the *rab2*^−/−^ mutant phenotype, we should observe presynaptic protein aggregation upon Golgi disruption. Depletion of the COPII complex protein Secretory 23 (Sec23), a component of the COPII vesicle coating (Fromme et al., 2008), caused a much stronger Golgi fragmentation than Rab2 depletion (Fig. 4 H), with an 87% increase in GM130 aggregate number (Fig. 4 I), 89% decrease of GM130 aggregate area (Fig. 4 J), and 43% decrease of GM130 intensity (Fig. 4 K). In clear contrast to *rab2*^−/−^ mutants, we could not observe any ectopic accumulations of presynaptic proteins (BRP, VGlut; Fig. 4, L–O) and no depletion of synaptic proteins (BRP, VGlut) at the synaptic terminal (Fig. 4, P–U). Additionally, depletion of the COPI complex protein ADP-ribosylation factor 79 (Arf79; Donaldson et al., 2005; Popoff et al., 2011) affected Golgi architecture and caused a twofold reduction in GM130 intensity, while GM130 aggregate number and area were not affected (Fig. S6 G). Again, presynaptic proteins (BRP, VGlut) did not aggregate in the somata of Arf79 depleted neurons, although overall BRP and VGlut levels were slightly increased (Fig. S6, H and I). Second, we asked if other than presynaptic proteins were also ectopically aggregating in Rab2-depleted somata and hence analyzed the transsynaptic membrane glycoprotein Neuroglian (Nrg) and the early endosomal marker Rbsn-5. Nrg protein remained bound to the plasma membrane in *rab2*^−/−^ mutant somata and did not aggregate into ectopic BRP accumulations, although the overall protein level was increased (Fig. 4, V and W). Nrg protein levels at the synaptic terminals were in fact increased, in contrast to the synaptic proteins being depleted in *rab2*^−/−^ mutants (Fig. 4, X–Z). In *rab2*^−/−^ somata labeled with Rbsn-5, uncommonly large Rbsn-5–positive organelles formed, which, however, were not copositive for BRP (Fig. S7, A–C). They likely represent enlarged early endosomes reflecting increased fluxes from the Golgi to early endosomes. Consistently, Rbsn-5 levels at Rab2-deficient terminals were not altered (Fig. S7, D and E). Thus, in the absence of Rab2, nonsynaptic proteins are still targeted to their membrane destination, and sorting from the Golgi is apparently not compromised, confirming that Rab2 specifically regulates presynaptic protein sorting from the trans-Golgi.

**Figure 5.**
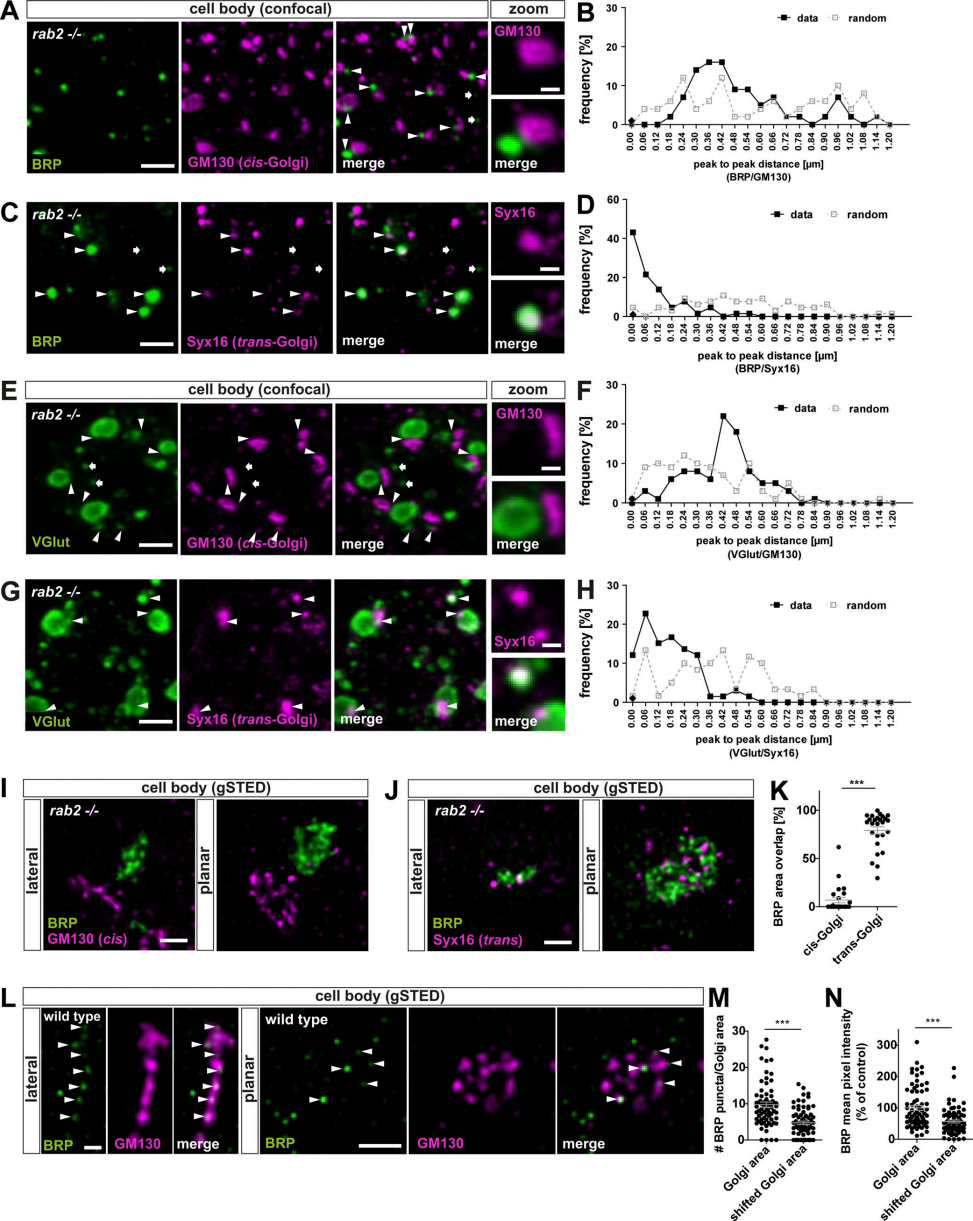
**Presynaptic precursors are derived from the trans-Golgi network. (A, C, E, and G)** Confocal images of neuronal cell bodies of *rab2*^−/−^ mutant animals costained for BRP (green) and GM130 (A and E; magenta) or Syx16 (C and G; magenta) with arrowheads indicating adjacent localizations (A and E) overlapping signals and arrows nonadjacent/overlapping events (C and G). **(B, D, F, and H)** Frequency of peak-to-peak distance distribution of BRP-positive accumulations and cis- or trans-Golgi. Black line for data and dashed gray line for 80 pixel–shifted images for random distribution with distance. For B, D, and F, percentage data/percentage random: 0.00 nm, 0/0, 43/4, 0/1, 12/1; 0.06 nm, 0/4, 21/0, 3/9, 22/13; 0.12 nm, 0/4, 13/4, 1/10, 15/1; 0.18 nm, 2/6, 4/3, 6/9, 16/5; 0.24 nm, 7/12, 7/9, 8/12, 13/10; 0.30 nm, 14/4, 1/6, 8/10, 12/8; 0.36 nm, 16/6, 4/7, 6/9, 1/10; 0.42 nm, 16/12, 0/10, 22/7, 1/13; 0.48 nm, 9/2, 1/7, 18/3, 3/3; 0.54 nm, 9/2, 1/7, 8/10, 1/11; 0.60 nm, 5/4, 0/9, 5/3, 0/10; 0.66 nm, 7/6, 0/3, 5/1, 0/3; 0.72 nm, 2/2, 0/7, 3/5, 0/3; 0.78 nm, 2/4, 0/4, 0/1, 0/1; 0.84 nm, 0/6, 0/4, 1/0, 0/3; 0.90 nm, 2/6, 0/6, 0/0, 0/0; 0.96 nm, 7/10, 0/0, 0/0, 0/0; 1.02 nm, 2/4, 0/0, 0/0, 0/0; 1.08 nm, 0/8, 0/0, 0/0, 0/0; 1.14 nm, 2/2, 0/1, 0/1, 0/0; 1.20 nm, 0/0, 0/1, 0/0, 0/0; *n* = 84 (B), *n* = 65 (D), *n* = 94 (F), and *n* = 96 (H). **(I, J, and L)** STED images of *rab2*^−/−^ mutant brains costained for BRP (green) and GM130 (I and L; magenta) or Syx16 (J; magenta) in lateral or planar view of the Golgi. Arrowheads point at Golgi-localized precursors. **(K)** Area overlap of BRP with either cis- or trans-Golgi (cis-Golgi: 6.87 ± 2.82%, *n* = 25; trans-Golgi: 79.08 ± 3.74%, *n* = 26). **(M)** Number of precursors within the Golgi area (Golgi area: 9.76 ± 0.71; shifted Golgi area: 4.74 ± 0.46, *n* = 72). **(N)** BRP mean pixel intensity within the Golgi area compared with random shifted GM130 channel (Golgi area: 100.0 ± 7.62; shifted Golgi area: 56.72 ± 4.76, *n* = 72). Scale bars: (A, C, E, and G) overview, 2 µm, zoom 0.5 µm; (I and J) 0.5 µm; and (L) lateral, 0.2 µm, planar 0.5 µm. All data provided as mean ± SEM. N represents single peak-to-peak measurements. Data from three VNCs (B, D, F, and H), and single Golgis of three VNCs (K, M, and N). ***, P < 0.001. gSTED, gated stimulation emission depletion.

We next analyzed the subcellular localization of ectopic BRP-positive aggregates in relation to the Golgi in *rab2*^−/−^-deficient somata and colabeled with a cis-Golgi (GM130) or trans-Golgi (Syx16) marker. Interestingly, BRP accumulations localized in the majority adjacent to the cis-Golgi (Fig. 5 A, arrowheads), with peak-to-peak distances of typically only around 400 nm (Fig. 5 B), and overlapped with the trans-Golgi (Fig. 5, C and D). We correspondingly observed a similar distribution of RIM-BP– (Fig. S7, F–I) and VGlut-positive (Fig. 5, E–H) aggregates with relation to both Golgi markers and could confirm these findings by STED microscopy. BRP accumulations also, under elevated magnification, formed adjacent to the cis-Golgi consistently both in lateral and planar views (Fig. 5 I) and clearly localized to the trans-Golgi area (Fig. 5 J; quantified in Fig. 5 K).

The trans-Golgi is the site of protein and lipid sorting and export for post-Golgi destinations (Keller and Simons, 1997; Klumperman, 2011; Pols et al., 2013). Assuming that in an early step of precursor biogenesis, synaptic proteins could sort in a Rab2-dependent process from the trans-Golgi, we should be able to detect precursors at the Golgi of wild-type neurons. Indeed, we observed BRP-positive puncta at the Golgi of wild-type somata (Fig. 5 L, arrowheads). Their numbers were higher at the Golgi compared with random-shifted Golgi areas in the cytoplasm, as well as BRP mean pixel intensity (Fig. 5, M and N). Notably, we also observed BRP-positive puncta in the cytoplasm (Fig. 5 L), and it is tempting to speculate that they might represent post-Golgi precursors ready to be exported from the cell body.

Thus, accumulating presynaptic proteins at the trans-Golgi of *rab2*^−/−^ mutant somata could represent immature precursors, interrupted in their biosynthetic maturation process due to loss of the membrane trafficking regulator Rab2.

### Tubule-shaped vesicular membranes accumulate at the trans-Golgi in ***rab2***^**−/−**^ mutants

**Figure 6.**
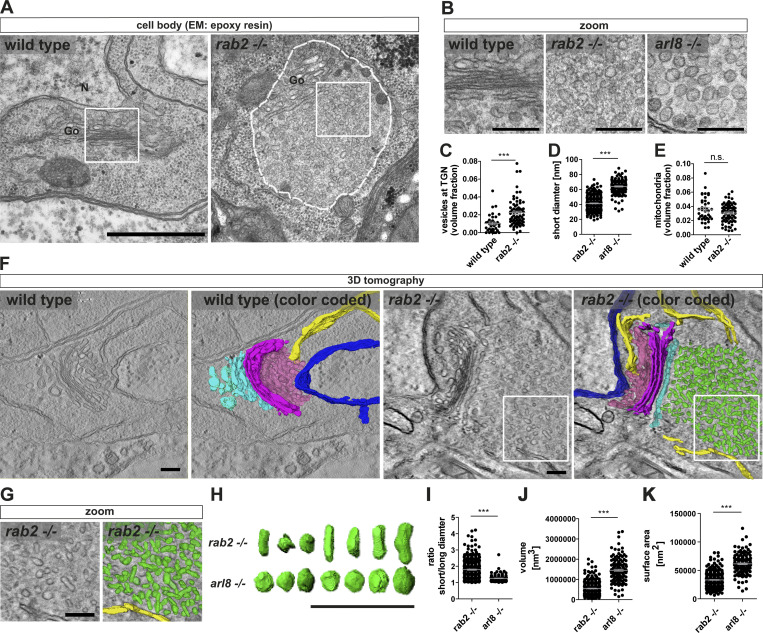
**Tubule-shaped vesicles accumulate at the trans-Golgi in *rab2****^**−/−**^*** mutants.** **(A)** Electron micrographs of wild-type and *rab2*^−/−^ mutant VNC somata containing Golgi (Go) and accumulation of immature presynaptic precursors at the trans-Golgi (dashed line). N, nucleus; white squares, zoom area. **(B)** Zoom of wild-type (left), *rab2*^−/−^ (middle), and *arl8*^−/−^ (right) mutant VNC somata. **(C)** Volume fraction of vesicles at the trans-Golgi network (TGN; wild-type: 0.0103 ± 0.0016, *n* = 39; *rab2*^−/−^**: **0.0224 ± 0.0018, *n* = 73). **(D)** Short precursors diameter of *rab2*^−/−^ and *arl8*^−/−^ mutants (*rab2*^−/−^: 41.38 nm ± 0.51, *n* = 313; *arl8*^−/−^: 64.06 nm ± 0.8, *n* = 138). **(E)** Mitochondria volume fraction (wild-type: 0.036 ± 0.003, *n* = 39; *rab2*^−/−^: 0.031 ± 0.002, *n* = 73). **(F)** Transmission EM tomography reconstruction of the Golgi area of wild-type and *rab2*^−/−^ mutants. Nucleus, dark blue; ER, yellow, ERGIC, pink; cis-Golgi, magenta; trans-Golgi, cyan; and immature precursors, green. White squares, zoom area. **(G)** Zoom. **(H)** Representative random selection of 3D reconstructed *rab2*^−/−^ and *arl8*^−/−^ mutant precursors. **(I–K)** Averaged *rab2*^−/−^ and *arl8*^−/−^ mutant vesicle short/long diameter ratio (*rab2*^−/−^: 1.79 ± 0.03, *n* = 313; *arl8*^−/−^: 1.26 ± 0.02, *n* = 138 (I); volume (J; *rab2*^−/−^: 0.56 ×10^6^ nm^3^ ± 21,040, *n* = 313; *arl8*^−/−^: 1.44 ×10^6^ nm^3^ ± 48,906, *n* = 138); surface area (K; *rab2*^−/−^: 33,563 nm^2^ ± 794, *n* = 313; *arl8*^−/−^: 60,795 nm^2^ ± 1,508, *n* = 138). Scale bars: (A) 500 nm, (B) 100 nm, (F and G) 200 nm, and (H) 300 nm. All data provided as mean ± SEM. N represents single vesicles (D, I, J, and K) or sections (C and E) of one or two VNCs. ***, P < 0.001. wt, wild-type. ERGIC, ER–Golgi intermediate compartment.

**Figure S8.**
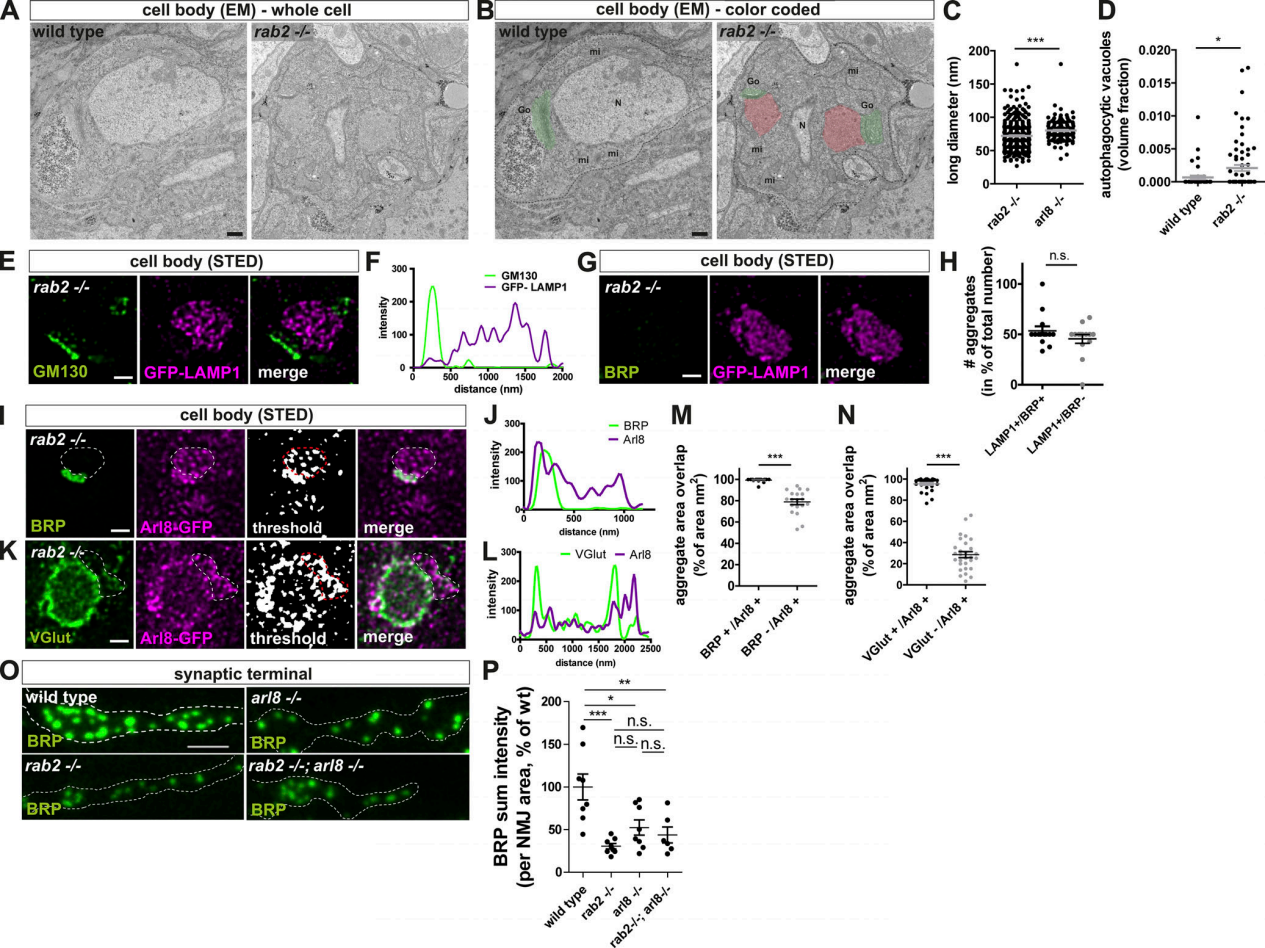
**Tubule-shaped vesicular membranes accumulate at the trans-Golgi in *rab2*^−/−^ mutants; LAMP1 and Arl8 distribution on presynaptic precursors.** **(A and B)** Colored: Electron micrographs of wild-type and *rab2*^−/−^ mutant whole cell bodies containing Golgi (Go) areas (green) with vesicle accumulations (red) at the trans-Golgi in *rab2*^−/−^ deficient neurons (N, nucleus; mt, mitochondria). Scale bar, 500 nm. **(C)** Quantification of long diameter of accumulating vesicles in *rab2*^−/−^ and *arl8*^−/−^ mutants (*rab2*^−/−^: 72.07 nm ± 1.27, *n* = 313; *arl8*^−/−^: 78.70 nm ± 1.54, *n* = 138). *n* represents single vesicles. **(D)** Volume fraction of autophagocytic vacuoles in wild-type and *rab2*^−/−^ mutants (wild-type: 0.0007 ± 0.0003, *n* = 39; *rab2*^−/−^ 0.0021 ± 0.0005, *n* = 73). **(E and G)** STED imaging of a representative precursor accumulations in *rab2*^−/−^ mutant somata immunostained for GFP-LAMP1 (magenta) and GM130 (E; green) or BRP (G; green). A fraction of the LAMP1-positive aggregates were not copositive for BRP. Scale bar, 0.5 µm. **(F)** Line profile of E. **(H)** Quantification of number of LAMP1-positive aggregates copositive for BRP or without BRP label in percentage of total number of aggregates (LAMP1-only positive aggregates: 46.01 ± 4.35%, *n* = 25; LAMP1- and BRP-positive aggregates: 53.99 ± 4.35%, *n* = 28). *n* represents single aggregates from three larvae and four or five VNC images/animal. **(I–N)** Arl8 localizes to BRP-positive and VGlut-positive aggregate areas. **(I and K)** STED imaging of a representative immature precursor accumulation in *rab2*^−/−^ mutant somata coexpressing Arl8-GFP immunostained for BRP (I; green) or VGlut (K; green) and Arl8-GFP (magenta). Threshold black-and-white image for a better visualization of the very weak Arl8 signal. Dotted lines mark the Arl8-positive area likely copositive for VGlut (I) and BRP (K). Scale bar, 0.5 µm. **(J and L) **Corresponding line profiles for I and K. **(M)** Quantification of aggregate area from I. BRP^+^/Arl8^+^ is the percentage of BRP-positive aggregate area (nm^2^) copositive for Arl8, and BRP^−^/Arl8^+^ is the percentage of total aggregate area (nm^2^) labeled with Arl8 and devoid of BRP (BRP^+^/Arl8^+^: 99.34 ± 0.39%, *n* = 19; BRP^-^/Arl8^+^: 78.90 ± 2.58%, *n* = 19). **(N)** Quantification of aggregate area from K. VGlut^+^/Arl8^+^ is the percentage of VGlut-positive aggregate area (nm^2^) copositive for Arl8, and VGlut^-^/Arl8^+^ is the percentage of total aggregate area (nm^2^) labeled with Arl8 and devoid of VGlut (VGlut^+^/Arl8^+^: 95.37 ± 1.12%, *n* = 29; VGlut^−^/Arl8^+^: 28.54 ± 2.83%, *n* = 29). **(O)** Confocal images of the synaptic terminal stained for BRP (green). Scale bar, 3 µm. **(P)** Quantification of the BRP sum intensity (wild-type: 100 ± 15.22%, *n* = 8; *rab2*^−/−^: 30.56 ± 3.18%, *n* = 8; *arl8^−/−^*: 52.43 ± 8.86%, *n* = 8; *rab2*^−/−^, *arl8*^−/−^: 43.74 ± 9.36%, *n* = 8) of O. All graphs show mean ± SEM. *n* represents single SVs (C) or sections (D) from one or two VNCs, single optical slices (one to four) of VNCs (H; total of three VNCs used), single aggregates from three VNCs (M and N), or single NMJs with one or two NMJs per larva (P). Normality was tested with the D’Agostino and Pearson omnibus normality test. If not normally distributed, the nonparametric Mann–Whitney test (C, D, H, M, and N) or Kruskal–Wallis test with Dunn’s multiple comparison test (P) was used. *, P < 0.05; **, P < 0.01; ***, P < 0.001. wt, wild-type.

For a detailed evaluation of the presynaptic protein accumulations in *rab2*^−/−^ mutants, we performed ultrastructural analysis by EM. Electron micrographs showed a striking accumulation of circular- to oval-shaped vesicular membranes at the trans-Golgi (Fig. 6, A and B; and Fig. S8, A and B), correlating with the position of presynaptic protein accumulations detected by light microscopy. No vesicle accumulations of this kind were observed at the Golgi in wild-type cells (Fig. 6, A and B; and Fig. S8, A and B), and the volume fraction of trans-Golgi network–associated vesicles of *rab2*^−/−^ mutants compared with wild type was significantly increased (Fig. 6 C).

But how do these maturation-arrested, immature precursors compare with the PLVs we described previously to accumulate in the cell body of *arl8*^−/−^ mutants (Vukoja et al., 2018)? The direct comparison of *arl8*^−/−^ mutant and *rab2*^−/−^ mutant electron micrographs revealed that PLVs detected in the absence of Arl8 were, with a ∼60-nm short diameter, significantly larger than *rab2*^−/−^ mutant precursors at ∼40 nm, possessed a more electron dense core (Fig. 6, B and D), and had a clearly more uniform, circular shape. Long diameters of both mutants were similar at ∼75 nm (Fig. S8 C). Notably, the volume fraction taken by mitochondria (Fig. 6 E) was not altered in Rab2-deficient neurons, in agreement with the confocal data (Fig. S1, Q and R), again showing that absence of Rab2 does not affect anterograde transport generically. Interestingly, we detected a threefold increase in the incidence of somatic structures resembling autophagocytic organelles in *rab2*^−/−^ mutants (Fig. S8 D), potentially interesting in the context of the previously described Rab2 function in the autophagic degradative pathway (Ding et al., 2019; Fujita et al., 2017; Lőrincz et al., 2017; Lund et al., 2018).

We performed 3D electron tomography to evaluate more deeply the apparent shape differences of precursor vesicles in *rab2*^−/−^ and *arl8*^−/−^ mutants. Clearly, immature precursors in *rab2*^−/−^ mutants localized, in agreement with the confocal and STED light-microscopic data (Fig. 5, A–H), along the entire trans-Golgi, while the cis-Golgi facing the nucleus was devoid of such vesicular accumulations (Fig. 6 F). Immature precursors were detectable now in the 3D reconstruction as heterogeneous vesicular or short tubular structures (Fig. 6, G and H), with an average short/long diameter ratio of ∼1.8 (Fig. 6 I). By contrast, PLVs accumulating in *arl8*^−/−^ mutants were rather homogenously shaped, circular vesicles with a short/long diameter ratio close to 1 (Fig. 6, H and I). Consequently, immature precursors of *rab2*^−/−^ mutants were threefold smaller compared with those of *arl8*^−/−^ mutants (Fig. 6 J) and had a twofold smaller surface area (Fig. 6 K).

In summary, we suggest that the 40-nm-size, tubule-shaped, clear-core vesicles forming at the trans-Golgi of *rab2*^−/−^ mutants represent immature and biosynthetically early arrested presynaptic precursors.

### Convergence of Golgi “trafficking routes” during maturation of presynaptic precursors

**Figure 7.**
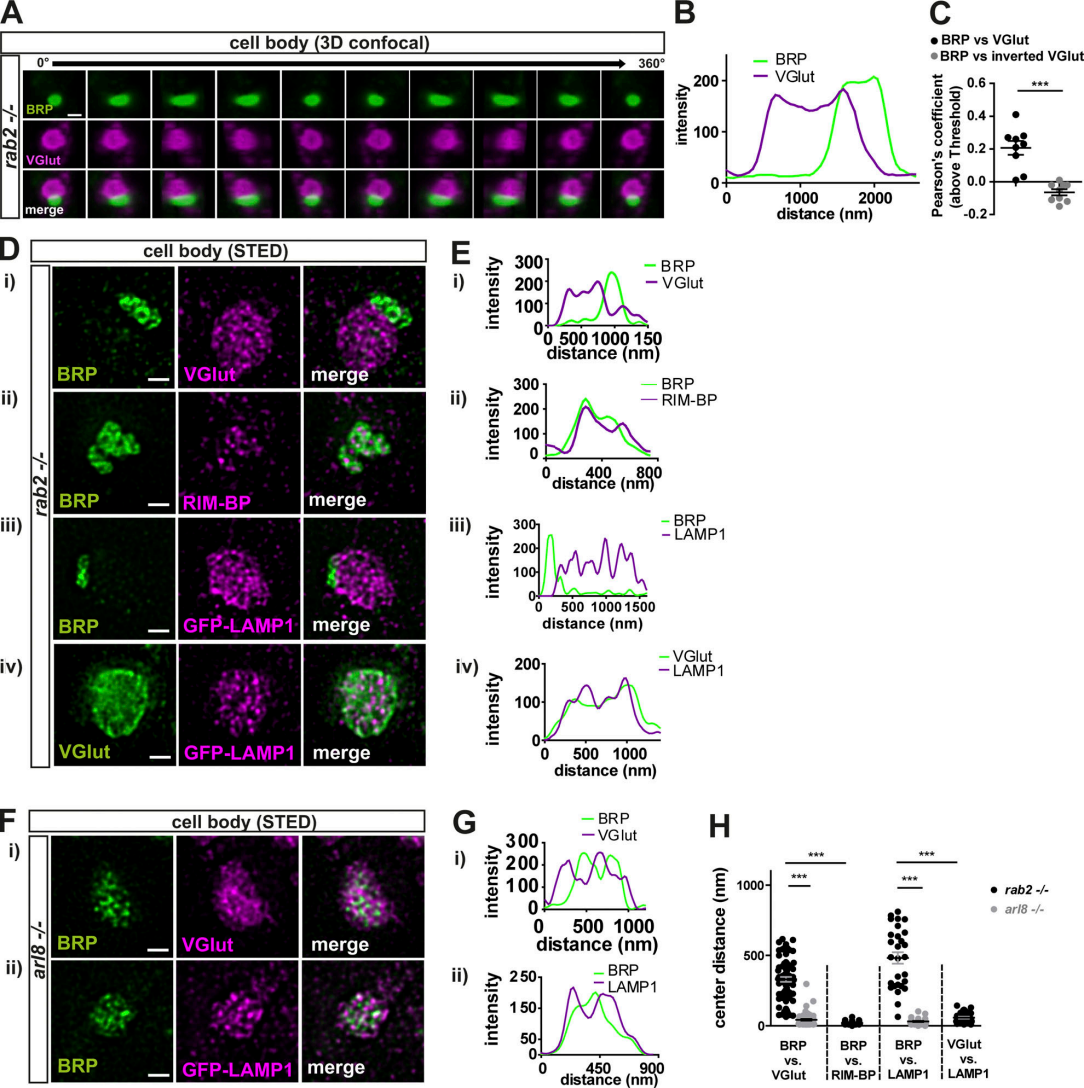
**Convergence of Golgi “trafficking streams” during precursors maturation.** **(A)** 3D reconstruction of confocal images of one precursor-accumulation labeled for BRP (green) and VGlut (magenta) with a 360° horizontal rotation. **(B and C)** Line profiles (B) and Pearson’s correlation coefficients (C; BRP versus VGlut: 0.21 ± 0.04, *n* = 9; inverted VGlut: −0.06 ± 0.02, *n* = 9). **(D and E)** STED imaging of precursor accumulations in *rab2*^−/−^ mutant somata labeled for BRP (green) and VGlut (Di; magenta), RIM-BP (Dii; magenta), GFP-LAMP1 (Diii; magenta), and VGlut (green) and GFP-LAMP1 (Div; magenta). **(Ei–iv)** Line profiles. **(F and G)** STED imaging of a representative precursor accumulation in *arl8*^−/−^ mutant somata labeled for BRP (green) and VGlut (Fi; magenta), and GFP-LAMP1 (Fii; magenta). **(Gi and Gii)** Line profiles. **(H)** Quantification of the area center distances. BRP versus VGlut: *rab2*^−/−^: 241.6 ± 19.00 nm, *n* = 41; *arl8*^−/−^: 30.85 ± 4.03 nm, *n* = 34. BRP versus RIM-BP: *rab2*^−/−^: 20.81 ± 2.95 nm, *n* = 24. BRP versus LAMP1: *rab2*^−/−^: 482.0 ± 40.62 nm, *n* = 28; *arl8*^−/−^: 31.53 ± 4.20 nm, *n* = 30. VGlut versus LAMP1: *rab2*^−/−^: 57.98 ± 6.93 nm, *n* = 31. BRP versus Arl8: *rab2*^−/−^: 60.97 ± 14.16 nm, *n* = 28. Scale bars: (A) 1 µm and (D and F) 0.5 µm. All data provided as mean ± SEM. *n* represents single evaluated precursor accumulation of three to five VNCs (C). ***, P < 0.001.

The confocal microscopy analysis of ectopic presynaptic protein aggregates forming in *rab2*^−/−^ mutants had shown that not all investigated proteins overlapped with the BRP signal (Fig. 1), suggesting that variable Golgi-sorting routes for different presynaptic proteins might exist. Hence, we performed 3D confocal image reconstruction to scrutinize the heterogeneous presynaptic protein distribution in more detail. A 360° rotation of a 3D reconstructed precursor field costained for BRP and VGlut demonstrated that BRP- or VGlut-positive precursors occupied contiguous instead of overlapping areas (Fig. 7 A), clearly visible in the line profiles (Fig. 7 B) and indicated by a low Pearson’s correlation coefficient (Fig. 7 C). STED imaging further underlined this finding: BRP-positive or VGlut-positive precursors occupied neighboring areas (Fig. 7 Di; and Fig. 7 Ei, for line profile), and quantification of the area center distance of both precursor fields showed a clear shift of several hundred nanometers (Fig. 7 H). By contrast, BRP- and RIM-BP–positive areas overlapped precisely (Fig. 7 Dii; and Fig. 7 Eii), and center distances between them were nearly zero (Fig. 7 H). Interestingly, GFP-LAMP1 preferentially localized to the VGlut-positive rather than the BRP-positive aggregate area (Fig. 7, Diii and Div; and Fig. 7, Eiii and Eiv), also evident in the large center distance between BRP and LAMP1 areas compared with VGlut and LAMP1 distances (Fig. 7 H). Consistently, GFP-LAMP1 accumulations localized in close proximity to the cis-Golgi (GM130) in *rab2*^−/−^ mutants (Fig. S8, E and F), similar to VGlut (Fig. 5 E). Notably, a subfraction of the ectopic LAMP1 accumulations in *rab2*^−/−^ mutants was devoid of BRP signal and not associated with early presynaptic precursor fields (Fig. S8, G and H), potentially related to the function of Rab2 in autophagic and lysosomal degradation (Ding et al., 2019; Fujita et al., 2017; Lőrincz et al., 2017; Lund et al., 2018). These data suggest that presynaptic proteins sort in a Rab2-dependent step from the trans-Golgi on two independent routes, separating AZ scaffold from SV proteins.

Interestingly, this segregation of presynaptic cargo was not observed in *arl8*^−/−^ mutants (Vukoja et al., 2018). We here applied STED microscopy on *arl8*^−/−^ mutants for comparability. Also, upon higher resolution, both VGlut and LAMP1 localized to the same area as BRP (Fig. 7, Fi and Fii; and Fig. 7, Gi and Gii) with a small center distances (Fig. 7 H), clearly contrasting with *rab2*^−/−^ mutants. Finally, we also expressed a GFP-tagged Arl8 (Vukoja et al., 2018) in *rab2*^−/−^ mutant animals and found that Arl8 was already present on both BRP- and VGlut-positive immature precursors (Fig. S8, I–L), as BRP- and VGlut-positive aggregates showed a nearly 100% overlap with Arl8 (Fig. S8, M and N). Collectively, these data suggest that in a first biosynthesis step, presynaptic proteins sort from the trans-Golgi on at least two distinct routes, a process regulated by the small GTPase Rab2. Immature precursors converge to larger and circular PLVs compatible with Arl8-dependent transport in subsequent maturation steps, characterized by a uniform presynaptic cargo load and a lysosomal membrane identity. This would suggest that Rab2 functions upstream of Arl8 during precursor biogenesis.

### Rab2 acts upstream of Arl8 in presynaptic precursor biogenesis

**Figure 8.**
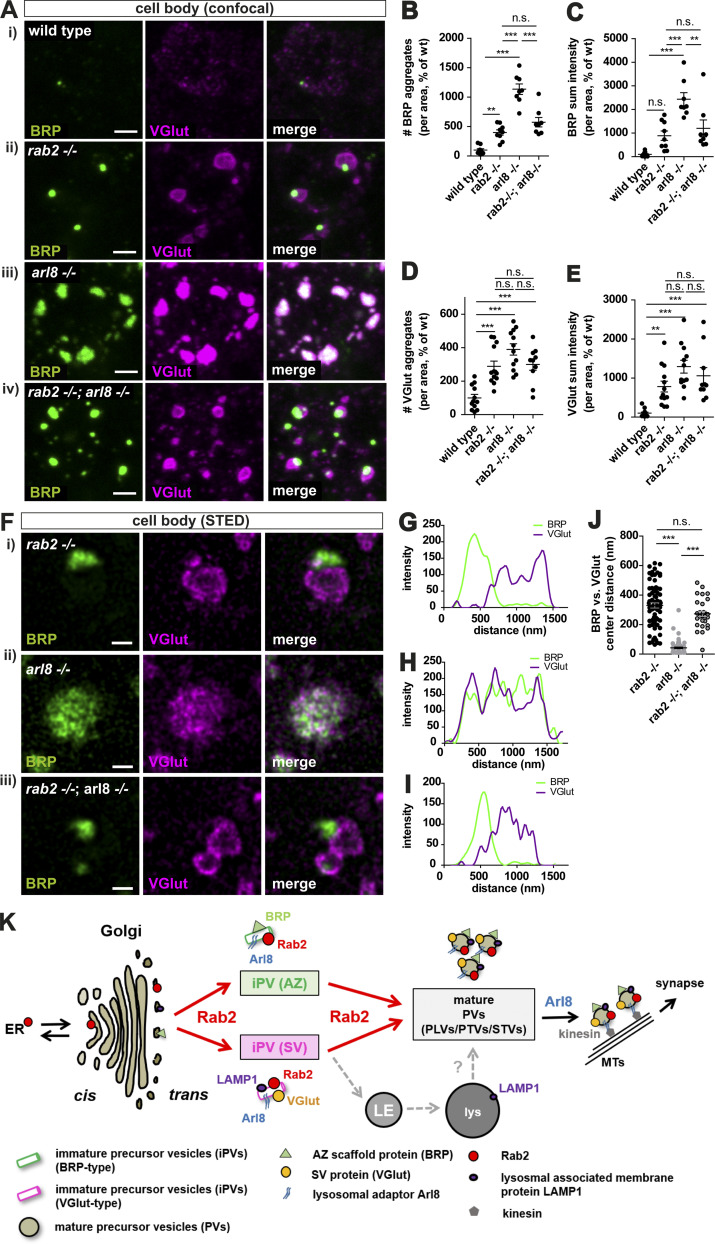
**Rab2 acts upstream of Arl8 in precursor biogenesis.** **(Ai–iv and Fi–iii)** Confocal images (A) and STED acquisitions (F) of the neuronal somata stained for BRP (green) and VGlut (magenta). **(B)** Number of BRP aggregates (wild-type: 100 ± 23.17%, *n* = 9; *rab2*^−/−^: 399.3 ± 44.69%, *n* = 9; *arl8*^−/−^: 1,134 ± 89.10%, *n* = 8; *rab2*^−/−^, *arl8*^−/−^: 573.9 ± 79.89%, *n* = 8). **(C) **BRP sum intensity (wild-type: 100 ± 32.66%, *n* = 9; *rab2*^−/−^: 889.8 ± 198.3%, *n* = 9; *arl8*^−/−^: 2,435 ± 276.1%, *n* = 8; *rab2*^−/−^, *arl8*^−/−^: 1,200 ± 355.5%, *n* = 8). **(D)** Number of VGlut aggregates (wild-type: 100 ± 20.77%, *n* = 12; *rab2*^−/−^: 288.9 ± 31.01%, *n* = 13; *arl8*^−/−^: 389.7 ± 34.03%, *n* = 12; *rab2*^−/−^, *arl8*^−/−^: 299.9 ± 34.26%, *n* = 10). **(E) **VGlut sum intensity (wild-type: 100 ± 28.84%, *n* = 12; *rab2*^−/−^: 781.3 ± 138.8%, *n* = 13; *arl8*^−/−^: 1,292 ± 163.9%, *n* = 12; *rab2*^−/−^, *arl8*^−/−^: 1,057 ± 211.4%, *n* = 10). **(G–I)** Corresponding line profiles for F. **(J)** Center distance between BRP- and VGlut-positive aggregates (*rab2*^−/−^: 329.8 ± 17.60%, *n* = 72; *arl8*^−/−^: 42.33 ± 5.55, *n* = 64; *rab2*^−/−^, *arl8*^−/−^: 273.2 ± 17.41, *n* = 31). **(K)** Model of presynaptic precursor biogenesis. Scale bars: (A) 2 µm and (F) 0.5 µm. All data are provided as mean ± SEM. *n* represents single VNCs (B–E) or single aggregates from three VNCs. **, P < 0.01; ***, P < 0.001. wt, wild-type. MT, microtubules. LE, late endosomes. PTV, Piccolo-Bassoon transport vesicles. STV, synaptic vesicle protein transport vesicles.

We created a *rab2^−/−^/arl8^−/−^* double mutant and compared the somatic presynaptic protein accumulation phenotype to the respective single mutants to access the epistatic relation of both proteins. Both number and intensity of BRP or VGlut accumulations were increased in *arl8*^−/−^ compared with *rab2*^−/−^ mutants (Fig. 8, A–E). Importantly, the *rab2^−/−^/arl8^−/−^* double-mutant larvae clearly resembled the phenotype of *rab2*^−/−^ single mutants, with quantifications detecting no significant differences between single *rab2*^−/−^ and *rab2^−/−^/arl8^−/−^* double mutants (Fig. 8, A–E). The segregation of BRP and VGlut within the ectopic accumulation of *rab2*^−/−^ mutants was clearly detectable in *rab2^−/−^/arl8^−/−^* double mutants, while *arl8*^−/−^ mutants again showed overlapping BRP and VGlut signals (Fig. 8 A). STED microscopy confirmed the BRP/VGlut segregation of *rab2^−/−^/arl8^−/−^* double mutants (Fig. 8, F–I), and quantification showed an equal center distance between *rab2*^−/−^ single and *rab2^−/−^/arl8^−/−^* double mutants, while single *arl8*^−/−^ mutants had a significantly reduced center distance (Fig. 8 J). Thus, disruption of precursor biogenesis in the absence of Rab2 prevents the entrance in a later Arl8-dependent biosynthetic maturation step, placing Rab2 upstream of Arl8. Consequently, when analyzing the synaptic terminals of *rab2^−/−^/arl8^−/−^* double mutants, no additive effect in the reduction of the synaptic material was observed (Fig. S8, O and P), consistent with our interpretation that both proteins indeed act in a sequential manner.

## Discussion

Although of fundamental importance, our understanding of the cell biological origin and molecular mechanics of presynaptic precursor biogenesis remains fragmentary. We here provide evidence that early precursor biogenesis is regulated by the conserved, Golgi-related small GTPase Rab2 (Chan et al., 2011; Lardong et al., 2015; Liu and Storrie, 2012; Lőrincz et al., 2017; Saraste, 2016; Tisdale and Balch, 1996). We suggest that precursor formation takes place in two consecutive steps: (1) sorting of precursor cargo proteins from the trans-Golgi on independent exit routes for AZ and SV proteins, (2) followed by a subsequent maturation step merging both exit routes. Convergence could occur either by direct fusion of early precursors or through conventional sorting at endo-lysosomal compartments, from which mature precursors are retrieved at a later time point (Fig. 8 K).

Several independent lines of evidence, including confocal, super-resolution light, and EM, live imaging, and genetic assays, support this model. Upon depletion of Rab2, biosynthetic presynaptic material accumulates ectopically at the trans-Golgi of motoneuronal somata, overlapping with the trans-Golgi marker Syx16 (Figs. 5 and 6). Interestingly, these accumulations did not show a uniform presynaptic material distribution but a segregation into two adjacent but not overlapping fields, positive for either presynaptic proteins (BRP, RIM-BP, UNC13) or SV proteins (VGlut, Syt-1; Figs. 1 and 7). These aggregates apparently do not represent degradative compartments as they were devoid of lysosomal and autophagosomal machinery markers, i.e., Cathepsin L, p62, and ATG8a (Figs. 1 and S1). Ultrastructural analysis revealed that ectopic accumulations consist of tubule-shaped, clear-core vesicles with a short diameter of ∼40 nm (Fig. 6). The elongated structures frequently appeared to consist of two to three attached spherical modules (Fig. 6 H), possibly consisting of immature precursors unable to proceed in a Rab2-dependent fusion or fission process. By contrast, the previously identified PLVs observed in *arl8*^−/−^ mutants (Vukoja et al., 2018) were uniformly circular-shaped with ∼60-nm diameters, a consequently larger volume, and a dense core (Fig. 6), apparently representing more matured precursors. Moreover, the matured precursor field of *arl8*^−/−^ mutants showed no segregation of AZ and SV proteins and is thus likely the result of a subsequent fusion step of the post-Golgi early precursors, either directly by mutual fusion or via the endo-lysosomal compartments (Fig. 8 K). As this fusion step evidently does not occur in the absence of Rab2, immature precursors here have lost the ability to fuse to mature vesicles. These findings would place Arl8 function downstream of Rab2 in the precursor biogenesis pathway and, indeed, we could show by genetic epistasis experiments that Rab2 acts upstream of Arl8 (Fig. 8).

Rab2 as a membrane-associated protein could function either directly at the trans-Golgi or on the arrested immature precursors by recruitment of Rab2 interaction partners to the nascent precursor membranes executing the subsequent fusion process, for example, SNARE proteins or proteins of the homotypic fusion and protein sorting complex, such as VPS39, already shown to be a direct interaction partner of Rab2 (Gillingham et al., 2014).

Interestingly, both the SV protein–positive early (*rab2*^−/−^ mutants) and the matured (*arl8*^−/−^ mutants) precursors are positive for the lysosomal marker LAMP1 (Fig. 7). Thus, the immature precursors already express some lysosomal membrane identity. LMPs traffic on nonclathrin-coated vesicles from the trans-Golgi network to the late endosome (Pols et al., 2013). It is tempting to speculate that LAMP1-positive early precursors might be related to these organelles. In contrast, Arl8, the lysosomal kinesin adaptor, localized to both types of immature precursors in *rab2* mutants (Fig. S5). Arl8, as a regulator of lysosome and lysosome-related organelle mobility (Khatter et al., 2015; Rosa-Ferreira and Munro, 2011), could confer motility to the mature precursors in the presynaptic precursor pathway by hooking them to the microtubule motor kinesin, as previously suggested in fly, mouse (Vukoja et al., 2018), and *Caenorhabditis elegans* (Klassen et al., 2010).

Downstream of somatic precursor biogenesis, Rab2 apparently remains associated, probably in its activated form, to the mature precursors as it trafficked anterogradely on BRP/Spinster-positive vesicles and effectively reached the synaptic terminal (Figs. 3 and S3). Retention of presynaptic proteins in the neuronal soma of *rab2*^−/−^ mutants consequentially caused a depletion of these proteins at the synaptic terminal and compromised presynapse formation and synaptic transmission (Fig. 2). The few synapses still forming remained roughly unaltered regarding architecture and SV number at the presynapse, suggesting a Rab2 function in the supply of presynaptic material rather than incorporation of precursor material into the assembling presynapse. However, as Rab2 is localized to the actively transported precursors (Fig. 3), it is conceivable that next to its upstream function in precursor biogenesis, Rab2 also directly contributes to the trafficking of presynaptic precursors.

Rab2 is described as a Golgi-related small GTPase implied in the bidirectional ER to Golgi trafficking (Liu and Storrie, 2012; Saraste, 2016; Tisdale and Balch, 1996), and indeed, we consistently observed subtle Golgi fragmentation defects in *rab2*^−/−^ mutants (Fig. 4, D–G), raising the possibility that generic Golgi sorting or protein biogenesis defects might be the primary cause of the *rab2*^−/−^ mutant phenotype. However, we could show that effective generic Golgi disruption did not phenocopy the *rab2*^−/−^ mutant phenotype, and other nonpresynaptic proteins were not accumulating at the trans-Golgi (Figs. 4 and S5), confirming a specific role for Rab2 in presynaptic precursor biogenesis.

The here-described function of Rab2 in precursor biogenesis could share mechanistic traits with the role of Rab2 and its effectors (RIC-19, RUND-1, CCCP-1, TBC-8) in DCV maturation in *C. elegans* (Ailion et al., 2014; Edwards et al., 2009; Hannemann et al., 2012; Sumakovic et al., 2009). DCVs store, transport, and release neuropeptides and neurotrophins through exocytosis. They form through secretion from the trans-Golgi network (Kim et al., 2006; Morvan and Tooze, 2008), where inadequate secretory proteins become incorporated into the immature DCVs, which are subsequently removed by clathrin-mediated membrane trafficking into the endo-lysosomal system (Ailion et al., 2014; Edwards et al., 2009; Kim et al., 2006; Morvan and Tooze, 2008; Sumakovic et al., 2009). Elegant studies in *C. elegans* showed that Rab2 and its effectors are required for the retention of neuropeptides in immature DCVs. Although we can only speculate, Rab2 function during the early phase of precursor biogenesis could comprise a similar mechanism by the retention or sorting of presynaptic and/or lysosomal proteins. Furthermore, future studies also need to evaluate a potential role of Rab2 in DCV maturation in *Drosophila*.

Our study contributes to a comprehensive model of the biosynthetic pathway underlying presynaptic precursor biogenesis and could support future research by connecting Rab2-related neurodevelopmental defects, e.g., memory defects in human (Li et al., 2015), autism spectrum disorders, or schizophrenia (Kiral et al., 2018; Takata et al., 2016), with the Golgi pathway–related neurodegenerative diseases (Rasika et al., 2018).

## Materials and methods

### Fly husbandry

*Drosophila melanogaster* strains were reared under standard laboratory conditions and raised at 25°C and 70% humidity on semi-defined medium (Bloomington recipe). For RNAi experiments, flies were kept at 29°C. For electrophysiological recordings, only male larvae were used. For all other experiments, both male and female animals were used. For genotypes and fly strains used, see Tables 1 and 2.

**Table 1.** Fly genotypes and strains

Fly strains	Source	Identifier
w^1118^ as wild-type	Bloomington *Drosophila* Stock Center	#3605
Bsc260 (Rab2 deficiency)	Bloomington *Drosophila* Stock Center	#23160
Rab2-Gal4-KO (#53)	Chan et al., 2011	
UAS-Rab2-YFP (wild-type)	Bloomington *Drosophila* Stock Center	#23246
Ok6-Gal4	Bloomington *Drosophila* Stock Center	#64199
UAS-dicer2	Vienna *Drosophila* Resource Center	#60008
UAS-Rab2-RNAi^105358^; UAS-Rab2-RNAi^34767^	Vienna *Drosophila* Resource Center	#105358 / #34767
genomic Rab2-GFP	Lund et al., 2018	
UAS-Rab2^Q65L^-YFP	Bloomington *Drosophila* Stock Center	#9760
UAS-BRP-D3-Straw	(UAS-BRP is a truncated BRP from aa 473–1227) Sigrist laboratory, Fouquet et al., 2009	
UAS-Spinster-RFP	Sweeney laboratory (University of York, UK)	
UAS-Sec23-RNAi	Vienna *Drosophila* Resource Center	#110568
UAS-Arf79F-RNAi	Bloomington *Drosophila* Stock Center	#29538
tub-GFP-LAMP1	Pulipparacharuvil et al., 2005	
PBac(RB)Gie ^e00336^ (for *arl8*^−/−^)	Bloomington Drosophila Stock Center	#17846
UAS-Arl8-GFP	Vukoja et al., 2018	

**Table 2.** Crosses and genotypes used

Crosses	Experiment
Rab2-Gal4-KO (#53) × Bsc260 (Rab2 deficiency)	Rab2 knockout (null mutant) (*rab2*^−/−^)
w^1118^	As wild-type control for mutant
Rab2-Gal4-KO (#53) × Bsc260; UAS-Rab2-YFP (wild-type)	Rab2 rescue (description, see below under "Rescue experiment")
Ok6-Gal4, UAS-dicer2 × UAS-Rab2-RNAi^105358^; UAS-Rab2-RNAi^34767^ Ok6-Gal4, UAS-dicer2 × w^1118^	Rab2 knockdown (RNAi) and control
Genomic Rab2-GFP	Rab2 localization (Figs. 3, 4, and S5)
Ok6-Gal4 × UAS-Rab2-YFP (wild-type)	In vivo (Fig. 3) Rab2 localization (Fig. S5)
Ok6-Gal4 × UAS-Rab2^Q65L^- YFP	In vivo (Fig. S4) Rab2 localization (Fig. S6)
Ok6-Gal4, UAS-BRP-D3-Straw × UAS-Rab2-YFP (wild-type)	In vivo (Fig. 3)
Ok6-Gal4, UAS-Rab2-YFP (wild-type) × UAS-Spinster-RFP	In vivo (Fig. 3)
Ok6-Gal4, UAS-BRP-D3-Straw × UAS-Rab2^Q65L^-YFP	In vivo (Fig. S5)
Ok6-Gal4, UAS-Rab2^Q65L^-YFP × UAS-Spinster-RFP	In vivo (Fig. S5)
UAS-dicer2 × UAS-Sec23-RNAi UAS-dicer2 × UAS-Arf79F-RNAi UAS-dicer2 × w^1118^	Golgi disruption and control (Figs. 4 and S6)
*rab2*^−/−^, tub-GFP-LAMP1	Fig. 7
PBac(RB)Gie e00336/PBac(RB)Gie ^e00336^	*arl8*^−/−^ mutant
*arl8*^−/−^, tub-GFP-LAMP1	Fig. 7
Bsc260; UAS-Arl8-GFP × Ok6-Gal4, Rab2-Gal4-KO (#53)	Fig. S8
Rab2-Gal4-KO (#53); PBac(RB)Gie ^e00336^ × Bsc260; PBac(RB)Gie ^e00336^	*rab2*^−/−^; *arl8*^−/−^ double mutant

### Rescue experiment

We used the genomic *rab2*^−/−^ mutant line (Chan et al., 2011), in which the Rab2 ORF was replaced by a Gal4, now transcriptionally regulated by the endogenous Rab2 transcription regulators, and thus expressed a UAS-Rab2-YFP at endogenous protein levels in the *rab2*^−/−^ mutant background (see also Tables 1 and 2).

### Immunostainings of larval neuromuscular junctions (NMJs) and brains

#### Immunohistochemistry for confocal and STED microscopy

For immunohistochemistry, dissections were performed in hemolymph-like solution 3 (HL3; Stewart et al., 1994; composition in millimolar: 70 NaCl, 5 KCl, 20 MgCl_2_, 10 NaHCO_3_, 5 trehalose, 115 sucrose, and 5 Hepes, pH adjusted to 7.2) by opening the third instar larvae dorsally along the midline and removing the entrails. Dissections were fixated with 4% paraformaldehyde in PBS (pH 7.2) for NMJ staining or Bouin’s fixative for staining of brain samples for 10 min. After fixation, the filets were washed with PBS plus 0.05% Triton X-100 and blocked for 60 min in 5% normal goat serum (Sigma-Aldrich; S2007). For immunostainings, the larvae were incubated with primary antibodies at 4°C overnight and subsequently washed in a PBS plus 0.05% Triton X-100 solution for 2 h at RT. Larvae were then incubated for 2–3 h with secondary antibodies at RT. Washing procedures were repeated. Larvae were finally mounted in Vectashield (Vector Laboratories), Mowiol (Sigma-Aldrich), or ProLong Gold (Thermo Fisher Scientific). Primary and secondary antibodies used in these studies are shown in Table 3.

**Table 3.** Primary and secondary antibodies

Primary antibodies	Source	Identifier
α-Tubulin (mouse, 1:10,000, used in WB only)	Thermo Fisher Scientific	62204
Atg8a, rat, 1:250	G. Juhasz laboratory, Eötvös Loránd University, Budapest, Hungary	N/A
ATP-synthase	Abcam	14748
BRP (Nc82), mouse, used 1:250	Developmental Studies Hybridoma Bank	Nc82
Cathepsin L, mouse, 1:200	R&D Systems	MAB22591
Dap160, rabbit, used 1:500	O. Shupliakov laboratory, Karolinska Institutet, Solna, Sweden	N/A
DOR/VPS18	H. Krämer laboratory, UT Southwestern, Dallas, TX	N/A
GFP (chicken, polyclonal, 1:2,000)	Abcam	ab13970
GFP (rabbit), 1:500	Invitrogen	A-11122
GFP (mouse), 1:500	Invitrogen	A-11120
GM130, rabbit, 1:500	Abcam	ab30637
p62, rabbit, 1:250	G. Juhasz laboratory	N/A
Rab2 (rabbit, 1:5,000, used in WB only)	Santa Cruz Biotechnology	sc-285667
Rbsn-5, rabbit, 1:1,000	A. Nakamura laboratory, RIKEN Center for Developmental Biology, Kobe, Japan	N/A
Rab7, rabbit, 1:1,000	A. Nakamura laboratory	N/A
RIM-BP^N-term^, rabbit, 1:500	S.J. Sigrist laboratory, Freie Universität Berlin, Berlin, Germany	9172
Synapse defective-1 (Syd-1), rabbit, 1:250	S.J. Sigrist laboratory	N/A
Synaptotagmin, rabbit, 1:1,000	BD Bioscience	N/A
UNC13A, guinea pig, 1:500	S.J. Sigrist laboratory	N/A
VGlut, rabbit, 1:500	H. Aberle laboratory, Düsseldorf, Germany	N/A
**Secondary antibodies**		
Alexa 488 (anti-rabbit, 1:500)	Invitrogen	A-11008
Alexa 488 (anti-mouse, 1:500)	Invitrogen	A-11001
Alexa 594 (anti-rabbit, 1:500 for STED)	Invitrogen	A32754
Alexa 594 (anti-mouse, 1:500 for STED)	Invitrogen	A32742
Atto 647N (anti-rabbit, 1:500 for STED)	Sigma-Aldrich	40839
Atto 647N (anti-mouse, 1:500 for STED)	Sigma-Aldrich	50185
Cy3 (anti-rabbit, 1:500)	Abcam	ab6939
Cy3 (anti-mouse, 1:500)	Abcam	ab97035
HRP-Alexa647 (conjugated antibody), 1:500	Jackson ImmunoResearch	123-605-021

N/A, not available; WB, Western blot.

#### Image acquisition and analysis

Conventional confocal and STED images were acquired with Leica DMI 6000 (SP8) and TCS SP8 gSTED 3× microscopes (Leica Microsystems), respectively. For confocal scans, an HC PL APO CS2 63× /1.40 NA oil objective (Leica Microsystems) was used; for STED, an HC PL APO CS2 100×/1.40 NA oil objective (Leica Microsystems) was used. Images were acquired at ∼20°C, and fluorochromes are indicated in the antibody section described above. Imaging medium was immersion oil (Sigma-Aldrich; 55822). Mounting medium was Mowiol 4-88 (Sigma-Aldrich). For signal detection, high-sensitive hybrid detectors with a 400–800-nm spectrum scanned for green and red channels and photomultiplier tubes with a 400–800-nm spectrum scanned for far red channels and were used for confocal scans. For STED, hybrid detector Sp GaAsP were used. Larval brain z-stacks had a step size of 0.3–0.5 µm between single optical slices. 40 z-slices of the central region along the dorsoventral axis were imaged. The NMJ z-stacks had a step size of 0.2–0.3 µm between single optical slices. All images were acquired using the LAS X software (Leica Microsystems). The ImageJ 1.52n software was used for the analyses of confocal and STED images. GraphPad Prism, v5.01, was used for statistical analyses. For STED microscopy, Huygens Deconvolution software was used applying a theoretical point spread function automatically computed based on pulsed- or continuous-wave STED-optimized function and the specific microscope parameters. Default deconvolution settings were applied. Images for figures were processed, if necessary, with ImageJ software to enhance brightness using the brightness/contrast function and smoothened (0.5-pixel Sigma radius) using the Gaussian blur function. Confocal stacks were processed with the image software Fiji (http://fiji.sc; Schindelin et al., 2012). Image analysis followed the standard protocol as described by Andlauer and Sigrist (2012b) and will be described in detail for the different analyses below.

##### NMJ and VNC quantifications

Type 1b NMJs on muscle 4 were analyzed. Zoom areas in the VNC were acquired of motorneuronal somata either lateral or central (dorsal) of the neutrophil. The HRP-Cy5 antibody signal was used as the template for a mask, restricting the quantified area to the shape of the NMJ. For larval brain aggregate quantification, a region of interest (ROI) was drawn via the freehand selection tool including only the cortex region of the larval VNC (sparing neuropil region and background). The original confocal stacks were converted to maximal projections (for brain, taking only the central 20 z-slices of the VNC without adjacent axons or weakly stained tissue in deeper layers), and after background subtraction, a mask of the synaptic area/brain aggregates was created by applying a manual threshold to remove the irrelevant lower-intensity pixels. The segmentation of single spots was done semi-automatically via the command “Find Maxima” embedded in the Fiji software and by hand with the pencil tool and a line thickness of 1 pixel. To remove high-frequency noise, a Gaussian blur filter (0.5-pixel Sigma radius) was applied. The processed picture was then transformed into a binary mask using the same lower threshold value as in the first step. This binary mask was then projected onto the original unmodified image using the “min” operation from the ImageJ image calculator.

The number of AZ or VNC aggregates in the graphs reflects the density of particles in both tissues and was normalized to either the NMJ (HRP) area or the freehand selection ROI of the cortex area. The AZ area (nm^2^) is the mean area value of all determined individual AZ area of one NMJ. For sum intensities “per NMJ area,” every single pixel intensity within the single spots/particles of the corresponding channel was first added up (calculated as “integrated density” per AZ/aggregate in Fiji), and subsequently all spot values per NMJ/cortex were summed up in order to estimate total protein levels of all particles within the respective tissue. Finally, this total sum was divided by the respective NMJ or cortex area to normalize these estimates for variations in imaged tissue size. *n* represents the number of NMJs/larval brains. For sum intensities “of NMJ,” protein mean pixel intensity within the HRP mask was calculated as “integrated density.” For protein mean pixel intensity “of AZ,” the mean pixel intensity of the individual AZs was determined and the mean value plotted. For protein mean intensity “of cortex area,” an ROI around the whole cortex area excluding the neuropile was created using the HRP signal as a reference. Mean pixel intensity of protein within this ROI was determined and the mean value plotted. The same procedure was applied for NMJ (“protein mean pixel intensity of NMJ”) using the HRP mask. All data were normalized to wild-type (100%). *n* for brain quantifications represents one VNC of one larva; four to eight larvae were analyzed. *n* for NMJs represents NMJs with one or two NMJs/animal and five or six animals/genotype.

##### Golgi (GM130) quantification of Golgi defects

Golgi quantifications were performed as described above regarding the basic image treatment. A stack of four to six slices from the VNC-zoom images was chosen and a maximum projection performed. Three to six clearly detectable cells were chosen for analysis and marked (ROI-area). Within this ROI, single GM130 spots were identified as described above, but without segmentation. The number of spots was divided by ROI-area and normalized to wild-type (100%). Area of the individual GM130 spot was determined, and the mean plotted, normalized to wild-type (100%). Mean pixel intensity of GM130 signal within the ROI-area was determined, and the mean plotted, normalized to wild-type (100%). *n* represents one VNC of one larva; four to eight larvae were analyzed.

##### Pearson’s correlation coefficient

For Pearson’s correlation coefficient, an image stack was acquired, and one focal plane was chosen for analysis after ROI selection within a single cortex soma or axonal bundle, respectively. ROIs were selected around aggregates with potential signal overlap of one to three aggregates in a range of 1.5–2.5 µm^2^. ImageJ Coloc_2 was used to determine the Pearson’s R value above threshold by testing the Rab2 channel against the other indicated second channel (BRP, VGlut, Spinster, etc.). As a control, the second channel was flipped horizontally (“inverted BRP, Spinster, VGlut, etc.”), and subsequently the Pearson’s R value above threshold was recalculated. This aims to show specific colocalization only in the original (not flipped/not inverted) overlapping channels and to exclude a general stochastic colocalization. Analyzed where overall 9–12 motoneuronal somata (one ROI per soma) of two or three VNCs, corresponding to two or three animals. For axonal colocalization, 10–15 axonal bundles (one ROI per axonal bundle) in three to five animals were selected.

##### BRP mean pixel intensity in boutons excluding AZs from STED analysis of NMJs

Bouton area was outlined manually (ROI-1), and outside bouton area was cleared. Gaussian blur with 2.0 was applied. A threshold of 30 was set and all AZs within this threshold marked (ROI-2). Subtraction of ROI-2 from ROI-1 (XOR function in Fiji ROI manager) and the new ROI was named ROI-3. In ROI-3 (bouton area without AZs), the mean pixel intensity was measured. Data were collected from two or three boutons of one NMJ per larva from three larvae.

##### Determination of the number of AZs per bouton area and AZ ring diameter from STED analysis of NMJs

For BRP ring diameter (C-terminal antibody Nc82) from STED analysis of NMJs, Nc82-positive AZs in a planar position with a minimum of three clusters/maxima were manually encircled, and ROIs were saved (1–14 AZs/bouton, three boutons/larvae, three larvae). ROI area was measured (Fiji) and diameter calculated assuming a circular ROI area according to the formula

$$\mathrm{d=}2\sqrt{\frac{A}{\pi }},$$

where *d* is the diameter and *A* is the area.

##### Area overlap measurement of BRP/VGlut and Arl8-GFP in percentage from neuronal somata

Single cells were manually chosen from three to five images/brain, three brains in total. Threshold values were set manually, and ROIs in both channels were outlined and the area measured. The AND function of the Fiji ROI manager was used to determine area overlap. Only the Arl8-positive area was determined by subtraction of the copositive area (AND; see above) from the total Arl8 area and percentages calculated.

##### Frequency determination in percentage of peak-to-peak distance of BRP-positive aggregates in rab2^−/−^ mutants and GM130-marked Golgi

We place line through BRP aggregates and Golgi, saved as ROI, used ROI manager MultiPlot tool, and determined peak-to-peak distance. For random shifted data, the BRP channel was shifted by 80 pixels. A cutoff at 1.2-µm distance was used, binned for 0.06 µm. We measured 1–14 distances from two VNC images per larva, with a total of three larvae analyzed.

##### Center distance measurements of STED-imaged presynaptic protein aggregates in rab2^−/−^ mutant somata

Single-channel images were created for individual aggregates. By defining a suitable threshold for each aggregate separately, a binary mask was created. Then, outlines of the aggregate were detected by the magical wand tool and subsequently saved as ROI. Using the ImageJ built-in measurement function “center of mass,” the respective x and y mass-central coordinates of each ROI (one per channel) were calculated. Distances of the centers of mass were calculated according to the function

$$\mathrm{d=}\sqrt{{\left[x\left(\mathrm{channel}1\right)-x\left(\mathrm{channel}2\right)\right]}^{2}+{\left[y\left(\mathrm{channel}1\right)-y\left(\mathrm{channel}2\right)\right]}^{2}},$$

(d = distance; x/y are the x/y coordinates). Experimental *n* represents the number of single aggregates tested in VNC images of two or three animals.

#### In vivo live imaging and analysis

In vivo imaging of intact *Drosophila* larvae was performed as previously described (Andlauer and Sigrist, 2012a). Briefly, third instar larvae were put into a drop of Voltalef H10S oil (Arkema) within an airtight imaging chamber. The larvae were anesthetized before imaging with 10 short pulses of a desflurane (UAS) air mixture until the muscles relaxed. Axons were imaged using confocal microscopy immediately after exiting the VNC.

Kymographs were plotted using a custom-written Fiji script (based on tsp050706.txt TimeSpacePlot [Kymograph] from Jens Rietdorf, downloadable at http://www.embl.de/eamnet/html/kymograph.html). In brief, temporal recordings of proximal axons (pixel size, 100 nm; *t* resolution, 0.8–1.7 s), expressing YFP/mRFP/strawberry-labeled proteins of interest, were background-subtracted, filtered via a Gausian blur (Sigma radius 0.5), and stabilized via the Fiji image registration plugin MultiStackReg. A time stack limited to the time frame of a single specific cotransport event was cropped from the original file. Sequentially, a segmented line ROI was drawn along the trajectory of the cargo (minimum of 30 s stable visibility). The final kymograph was (automatically) created by adding the line ROI (average width, 3–10 pixels) per time frame on top of the preceding line ROI in a concatenated fashion.

Quantification of (co)transport events in proximal axons was performed as follows. Per indicated coexpression genotype, two or three different axonal nerve bundles (5-min imaging time per axon) were imaged in five or six animals. Every file was screened for transport events by quantifying events of sufficient intensity and traceability (stable identification of the unidirectional cargo trajectory for at least 4 µm in one channel) via drawing a linear ROI starting from the point of first discovery to the end point in the last sufficiently recognizable section. The length of this ROI was divided by the time (number of traceable images of the single event × *t* resolution per file), and the resulting speed value (µm/s) was listed according to the cargo direction (anterograde/retrograde). Every ROI (created in channel one) was compared with the corresponding coexpression channel and only counted as cotransport if this second channel indicated a similar unipolar movement event in various images (at least three or four different temporal sections within the same time frame while indicating a sufficient signal overlap).

#### Electrophysiology

TEVC recordings were performed as previously reported (Matkovic et al., 2013). In short, spontaneous recordings (miniature excitatory junction currents [mEJCs], 90 s), single-evoked recordings (eEJCs, 20 repetitions at 0.2 Hz), and high-frequency recordings (PP 10-ms or 30-ms interstimulus interval, PP10 or PP30, 10 repetitions at 0.2 Hz) were recorded. All experiments were performed on third instar larvae raised at 25°C on semi-defined medium (Bloomington recipe). Dissection and recording medium was extracellular HL3 (Stewart et al., 1994; in millimolar: 70 NaCl, 5 KCl, 20 MgCl_2_, 10 NaHCO_3_, 5 trehalose, 115 sucrose, and 5 Hepes, pH adjusted to 7.2). Dissections were done in ice-cold Ca^2+^-free HL3 medium, while mEJC, eEJC, and high-frequency recordings were performed in 1.5 mM Ca^2+^ HL3 at room temperature. For all physiological recordings, intracellular electrodes with a resistances of 15−25 MΩ (filled with 3 M KCl) were placed at muscle 6 of abdominal segment A2/A3. Acquired data were subjected to a low-pass filter at 1 kHz and sampled at 10 kHz. Traces of both miniature and evoked postsynaptic currents were recorded in TEVC mode (AxoClamp 2B; Axon Instruments). The command potential for mEJC recordings was −80 mV, and it was −60 mV for all other recordings. Only cells with an initial membrane potential between −50 and −70 mV and input resistances of ≥4 MΩ were used for further analysis. eEJC and PP traces were analyzed for standard parameters (amplitude, rise time, decay, charge flow, PP ratio) by using a semi-automatic custom-written MATLAB script (Mathworks; v. R2009a). Stimulation artifacts in eEJC recordings were removed for clarity. mEJC recordings were analyzed with pClamp 10 software (Molecular Devices).

#### Western blot analysis of larval brains

Larval central nervous system (CNS) protein extractions were performed as follows: 10 CNSs were dissected from third instar larvae. The resulting tissues were sheared manually in 5 µl of 2% SDS aqueous solution using a micropistil secured into a 1.5-ml cup. Subsequently, 0.5 µl of a 10% Triton X-100 aqueous solution and 5 µl of 2× Laemmli sample buffer were added. Samples were then heated at 95°C for 10 min. Centrifugation was performed for 2 min at 16,000 *g*, in order to pellet debris. For larval CNS, 10 µl (equivalent to 10 larval CNS) was subjected to denaturing SDS-PAGE using a 6% Tris-HCl gel.

Proteins of the PAGE gel were then transferred onto a nitrocellulose membrane blocked with 5% skim milk in 1× PBS supplemented with 0.1% Tween-20 and probed with rabbit anti-Rab2 (1:5,000; Santa Cruz Biotechnology; sc-285667) and mouse anti-tubulin (1:100,000; Thermo Fisher Scientific; 62204) diluted in 5% milk in 1× PBS, supplemented with 0.1% Tween-20. After washing, secondary anti-rabbit or anti-mouse IgG HRP-conjugated antibodies (Dianova) were used for detection (Dianova) in conjunction with an enhanced chemoluminescence (GE Healthcare ECL Prime; RPN 2232) detection system with Hyperfilm ECL (GE Healthcare).

#### EM

Conventional embedding for transmission EM was performed as described previously (Matkovic et al., 2013). In brief, dissected third instar larvae were fixed with 4% PFA and 0.5% glutaraldehyde in 0.1 M dissolved in PBS for 10 min and subsequently in glutaraldehyde (2% glutaraldehyde in 0.1 M sodium cacodylate) for 60 min, then washed in sodium cacodylate buffer and postfixed with 1% osmium tetroxide and 0.8% KFeCn in 0.1 M sodium cacodylate buffer (1 h on ice). After washing with sodium cacodylate buffer and distilled water, the samples were stained with 1% uranyl acetate in distilled water. Samples were dehydrated, infiltrated in EPON resin, and subsequently muscles 6/7 of abdominal segment A2/3 were removed by dissection. Blocks, collected in an embedding mold, were polymerized and cut in 65–70-nm-thin serial sections. Sections were then postfixed and poststained with uranyl acetate/lead citrate. Micrographs were produced using a JEM 1011 microscope equipped with an Orius 1200A Gatan camera using the DigitalMicrograph software package (Gatan).

For quantification, the plasma membrane and the electron-dense T-bar were detected by eye and labeled manually. T-bar roof size was measured using a straight line connecting the furthest distance of the upmost T-bar dense material (in relation to the plasma membrane). T-bar area was obtained by surrounding the dense material and measuring the area of the created ROI.

For 2D morphometric analysis, images of neuronal soma from VNCs were imaged using a Zeiss 900 transmission EM. For 3D analyses, 250-nm sections were cut and collected on coated slotted grids with 10-nm gold fiducials. Once the Golgi was located, a series of images from +60° to −60° were taken with a 1° step using a Tecnai G20 microscope. Etomo/IMOD (Kremer et al., 1996; https://bio3d.colorado.edu/imod/) and Microscopy Image Browser (Belevich et al., 2016; http://mib.helsinki.fi/index.html) were used to work with 3D volumes and render 3D models of Golgi and Golgi-associated structures.

### Quantification and statistical analysis

Data were analyzed using GraphPad Prism v5.01. Normality was tested with the D’Agostino and Pearson omnibus normality test. If the sample size was too low for D’Agostino and Pearson omnibus normality test (*n* > 7), Gaussian distribution was assumed. If data were normally distributed (or assumed to be normally distributed for *n* < 7) and two groups were compared, an unpaired, two-tailed *t* test was used (Fig. 1, B, C, E, G, I, and K; Fig. 2, C–H, K, O, Q, and T–W; Fig. 3, M and Q; Fig. 4, F, G, I–K, M, O, R, T, and W; Fig. 6 E; and Fig. 7 C). If data were normally distributed (or assumed to be normally distributed for *n* < 7) and more than two groups were compared, a one-way ANOVA with Tukey’s post hoc test was used (Fig. 1, M and N; and Fig. 8, B–E). If data were not normally distributed and two groups were compared, a Mann–Whitney *U* test was used (Fig. 2, J, L, N, P, and R; Fig. 3, C, F, and I; Fig. 4, C, E, S, U, and Z; Fig. 5, K, M, and N; Fig. 6, C, D, and I–K; and Fig. 7 H). If data were not normally distributed and more than two groups were compared, the Kruskal–Wallis test with Dunn’s post hoc test was used (Fig. 8 J). For supplemental figures, see supplemental figure legends. Significance is noted by *, P < 0.05; **, P < 0.01; ***, P < 0.001. No statistical methods were used to predetermine sample sizes, but sample sizes in these experiments were similar to those generally employed in the field. Data collection and analyses were not performed blind to the conditions of the experiments, nor was data collection randomly. For electrophysiological recordings, genotypes were measured in an alternating fashion on the same day and strictly analyzed in an unbiased manner.

### Online supplemental material

Fig. S1 shows that presynaptic proteins accumulate in cell bodies of *rab2*^−/−^-deficient neurons. Fig. S2 shows presynaptic and nonsynaptic proteins in cell bodies of *rab2*^−/−^-deficient neurons. Fig. S3 shows that presynaptic Rab2 depletion mediated by RNAi knockdown caused accumulation of presynaptic proteins in neuronal cell bodies. Fig. S4 shows that presynaptic biogenesis relies on the Rab2-dependent delivery of presynaptic material. Fig. S5 shows that Rab2 is a component of anterogradely and retrogradely trafficking presynaptic precursors. Fig. S6 shows that Rab2 localizes to the Golgi in neuronal somata, and disrupted Golgi is not affecting presynaptic precursor biogenesis. Fig. S7 shows that Golgi sorting and targeting of nonsynaptic proteins are not affected, and RIM-BP accumulates at the trans-Golgi in *rab2*^−/−^ mutants. Fig. S8 shows that tubule-shaped vesicular membranes accumulate at the trans-Golgi in *rab2*^−/−^ mutants and shows LAMP1 and Arl8 distribution on presynaptic precursors.
